# Cu‐Catalyzed Aerobic Oxidative C─C Cleavage in Lignin‐Derived Oligomers and Biological Funneling of the Monomeric Products

**DOI:** 10.1002/anie.202515588

**Published:** 2025-11-17

**Authors:** Surajudeen Omolabake, Dillon T. Hofsommer, Kathryn M. Mains, Chad T. Palumbo, Davide Rigo, Allison Z. Werner, Gregg T. Beckham, Shannon S. Stahl

**Affiliations:** ^1^ Department of Chemistry University of Wisconsin‐Madison 1101 University Avenue Madison Wisconsin 53706 USA; ^2^ Wisconsin Energy Institute University of Wisconsin‐Madison Madison Wisconsin 53726 USA; ^3^ Renewable Resources and Enabling Sciences Center National Renewable Energy Laboratory Golden Colorado 80401 USA; ^4^ Center for Bioenergy Innovation Oak Ridge National Laboratory Oak Ridge Tennessee 37830 USA

**Keywords:** Aerobic oxidation, Catalysis, C─C bond cleavage, Lignin monomers, Lignin oligomer

## Abstract

Existing methods for lignin deconstruction to aromatic monomers primarily cleave carbon–oxygen bonds within the polymer, resulting in sub‐optimal monomer yields and formation of oligomers that retain intact carbon–carbon bonds. Here, we demonstrate that copper‐catalyzed aerobic oxidation under aqueous alkaline conditions promotes oxidative cleavage of carbon–carbon bonds in lignin oligomers derived from reductive catalytic fractionation (RCF) of pine and poplar biomass. Fundamental insights are gained from reactions of model compounds that resemble subunits present in RCF oligomers. Optimal results are achieved in a flow reactor that provides precise control over O_2_ delivery, temperature, and reaction residence time. The Cu‐catalyzed aerobic oxidation conditions access aromatic monomers in 19 and 34 wt% monomer yields, respectively, from pine‐ and poplar‐derived RCF oligomers. Overall, the sequence consisting of biomass RCF into monomers and oligomers followed by oxidative deconstruction of the RCF oligomers generates substantially higher yields of aromatic monomers from lignin. Engineered strains of *Pseudomonas putida* support biological funneling of the oligomer‐derived oxygenated aromatic compounds into *cis*,*cis*‐muconic acid from pine or 2‐pyrone‐4,6‐dicarboxylic acid from poplar.

## Graphical abstract

Catalytic aerobic oxidation under aqueous alkaline conditions promotes C–C cleavage in pine‐ and poplar‐derived lignin oligomers, accessing high yields of aromatic monomers. The monomeric phenols obtained from this process then undergo effective biological conversion into *cis*,*cis*‐muconic acid and 2‐pyrone‐4,6‐dicarboxylic acid using engineered strains of *Pseudomonas putida*.

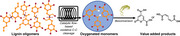

## Introduction

**Figure 1:**
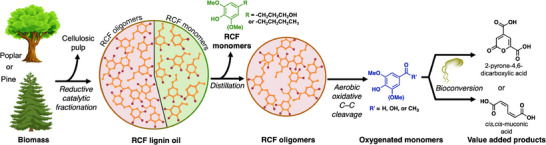
Alkaline aerobic oxidation performed on oligomers from the reductive catalytic fractionation (RCF) of pine and poplar biomass leads to oxidative monomers, which are converted to either muconic acid or 2‐pyrone‐4,6‐dicarboxylic acid (PDC), respectively.

Efforts to deconstruct lignin into aromatic chemicals have been the focus of extensive research.^[^ ^1^, ^2^, ^3^, ^4^, ^5^, ^6^, ^7^, ^8^, ^9^, ^10^, ^11^ ^]^ The yields from such processes are often limited by the cleavable bonds present in the lignin and strongly depend on the plant source (grasses, softwood, hardwood, etc.), lignin extraction method, and deconstruction process, among other factors. To date, most lignin deconstruction methods target carbon–oxygen (C─O) bonds or the weak carbon–carbon (C─C) present in β‐O‐4 linkages, and they generate a collection of aromatic monomers together with oligomeric byproducts that retain stronger C─C bonds between the aromatic subunits.^[^ ^12^, ^13^, ^14^ ^]^ Catalytic methods that could cleave the C─C bonds within these oligomers would lead to higher yields of lignin‐derived monomers and represent a major goal for lignin valorization.^[^ ^15^ ^]^ For example, reductive catalytic fractionation (RCF) is an efficient method to convert biomass into cellulosic pulp and a lignin oil that consists of aromatic monomers and C─C‐linked oligomers (Figure 1).^[^ ^7^, ^16^, ^17^, ^18^ ^]^ The oligomers are being explored for various applications,^[^ ^19^, ^20^, ^21^ ^]^ but they also represent a feedstock that is largely untapped for the production of additional aromatic monomers.

Methods for C─C bond cleavage in lignin and lignin‐derived oligomers have been the focus of recent attention.^[^ ^15^ ^]^ Notable success has been achieved recently through catalytic cracking/hydrogenolysis methods to afford hydrocarbon products,^[^ ^22^, ^23^ ^]^ while alternative approaches have focused on oxidation strategies.^[^ ^24^, ^25^, ^26^ ^]^ Samec and coworkers used a stoichiometric oxoammonium reagent, Bobbitt's salt (4‐acetamido‐2,2,6,6‐tetramethyl‐1‐oxo‐piperidinium tetrafluoroborate), to promote oligomer oxidation and hydrolysis, generating 2,6‐dimethoxybenzoquinone (DMBQ) in 18 wt% yield from birch RCF oligomers.^[^ ^24^ ^]^ We used catalytic autoxidation with a Co/Mn/Br catalyst system to promote oxidative C─C cleavage of poplar RCF oligomers. The phenols in the oligomers were first protected with acetyl groups to prevent inhibition of the radical autoxidation mechanism,^[^ ^27^ ^]^ and the process afforded acetylated vanillic acid, vanillin, syringic acid, and syringaldehyde as major products in 13 wt% yield.^[^ ^25^ ^]^ We subsequently reported a related Mn/Zr‐catalyzed autoxidation method to convert RCF oligomers from pine and poplar into monomers. In this case, the phenols were methylated prior to the reaction, and the major products consisted of methyl aryl ether derivatives of vanillic acid and vanillin from pine (23 wt% yield) and both syringyl‐ (S) and guaiacyl‐ (G) based monomers from poplar (18 wt% yield).^[^ ^26^ ^]^

These precedents for oxidative C─C cleavage highlight the opportunity to access aromatic monomers from RCF oligomers. The present study was initiated with the goal of identifying more practical conditions that could retain use of O_2_ as the oxidant but avoid the need for phenol protecting groups. Aqueous aerobic oxidation conditions widely studied for lignin deconstruction^[^ ^28^, ^29^, ^30^, ^31^, ^32^, ^33^, ^34^, ^35^, ^36^ ^]^ and delignification of pulp^[^ ^37^, ^38^, ^39^ ^]^ offer a potential strategy to achieve this goal. The Borregaard LignoTech process, which converts industrial lignosulfonates derived from softwood into vanillin,^[^ ^30^, ^40^, ^41^ ^]^ is a prominent example. Several of these aerobic oxidation methods not only tolerate free phenols, but they generate aromatic monomers in yields that exceed values expected from C─O bond cleavage. The greater than theoretical yields obtained from these methods imply that C─C cleavage takes place and that alkaline aerobic oxidation could be used to promote C─C cleavage in lignin‐derived oligomers.

Here, we evaluate Cu‐catalyzed aerobic oxidation conditions to convert RCF oligomers to aromatic monomers. Studies of model dimers that resemble subunits in RCF oligomers provide insights into the reactivity of different C─C bonds. Applying the same conditions to reactions of RCF oligomers from poplar and pine biomass demonstrates effective oxidative cleavage of C─C bonds, without requiring protection of the phenols. Use of a recently reported flow‐reactor^[^ ^36^ ^]^ significantly improves control over reaction temperature and time, enabling improved yields of aromatic monomers. The oxygenated aromatic product mixtures obtained from these reactions are shown to be effective feedstocks for biological funneling^[^ ^11^, ^42^, ^43^, ^44^, ^45^ ^]^ into *cis*,*cis*‐muconic acid or 2‐pyrone‐4,6‐dicarboxylic acid (PDC), two performance‐advantaged bioproducts,^[^ ^46^ ^]^ by using an engineered strain of the soil bacterium *Pseudomonas putida* KT2440 (Figure 1).^[^ ^46^, ^47^ ^]^

## Results and Discussion

### Synthesis and Reactivity of Model Dimers

**Figure 2:**
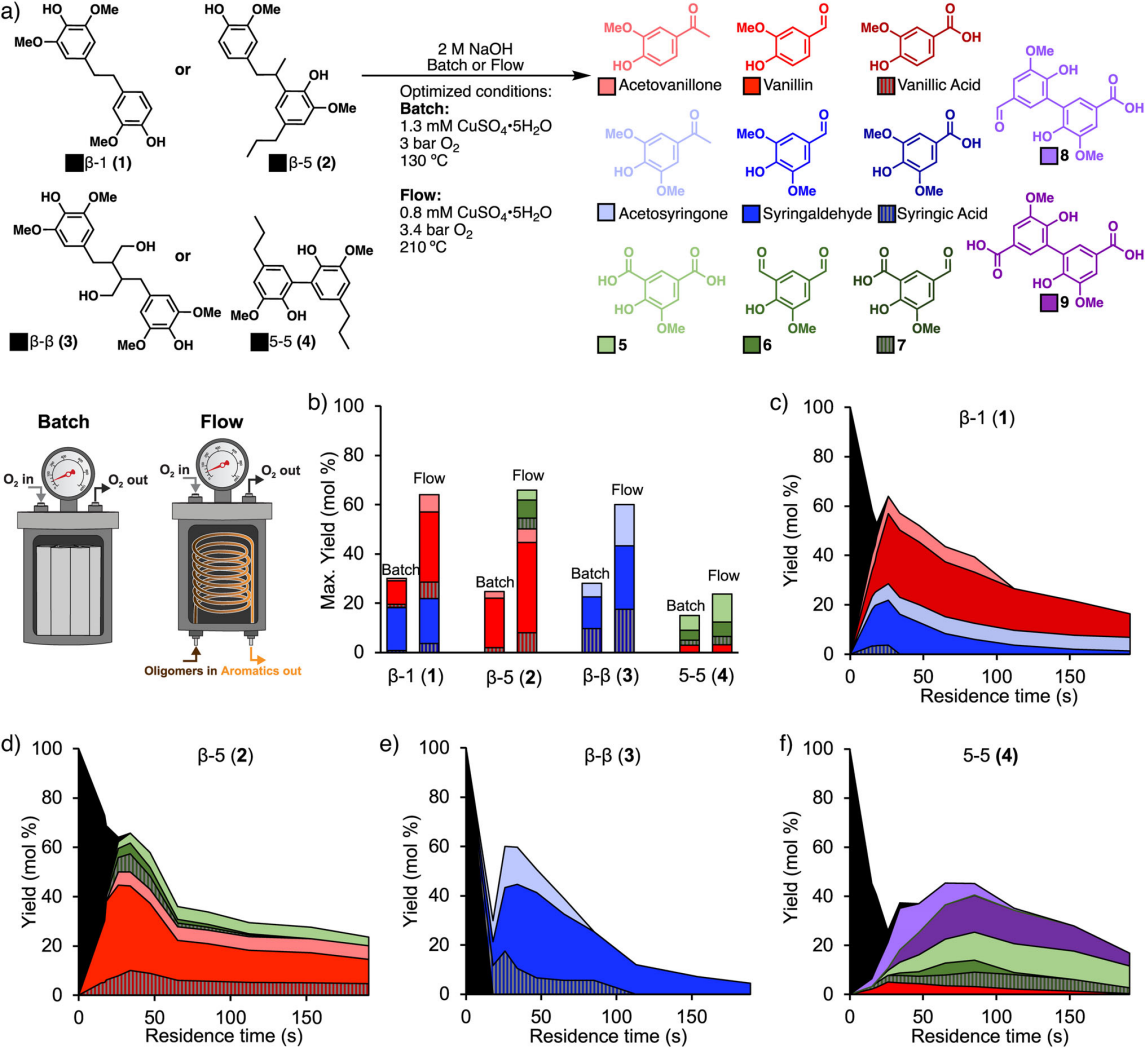
a) Aromatic monomers and dimers obtained from the oxidative and alkaline cleavage of C─C bonds in dimeric model compounds in batch and flow. b) The maximum monomer yields for batch and flow systems under optimized conditions. Time course data with monomer and dimer yield results for the cleavage of β‐1 model **1** (c), β‐5 model **2** (d), β‐β model compound **3** (e), and 5–5 model compound **4** (f). Dimer compound **8** is 5′‐formyl‐2′,6‐dihydroxy‐3′,5‐dimethoxy‐(1,1′‐biphenyl)‐3‐carboxylic acid, and dimer compound **9** is 6,6′‐dihydroxy‐5,5′‐dimethoxy‐(1,1′‐biphenyl)‐3,3′‐dicarboxylic acid.

To begin exploring C─C cleavage, several dimers with subunits that have been identified in RCF oil^[^ ^13^, ^14^ ^]^ were selected and synthesized according to literature procedures.^[^ ^48^, ^49^, ^50^, ^51^, ^52^, ^53^, ^54^ ^]^ Specific structures include dendrophenol **1** as a β‐1 derivative, the 1,2‐propenyl‐linked bisphenol **2** as a β‐5 derivative, the β‐β dimer model **3**, and a 5,5‐linked biaryl compound **4** (Figure 2a).

Initial studies of Cu‐catalyzed oxidation of these compounds used batch reaction conditions adapted from a previous study of poplar lignin deconstruction.^[^ ^32^ ^]^ Reactions were performed in a stainless‐steel pressure vessel with 3 mM substrate in 2 M NaOH. They were quenched after the designated reaction time by cooling to room temperature and venting the gas pressure. Conditions were varied to optimize the temperature (100–160 °C), O_2_ pressure (0–7 bar), reaction time (25–55 min; values include the heat‐up period), and CuSO_4_ loading (0.0–1.6 mM) (see Section 4, Table S1, and Figure S1 of the Supporting Information for details). Optimized batch conditions (1.3 mM CuSO_4_•5H_2_O, 3 bar O_2_, 130 °C, 37 min) led to complete conversion of substrates **1**–**3** with monomer yields of 30%, 25%, and 28%, respectively, distributed among syringyl and guaiacyl derivatives (Figure 2b; note: 100% yield accounts for two monomers from each dimeric substrate). The biaryl compound **4** reacts under similar conditions, affording a mixture of monomeric aromatics (vanillin and **5**–**7**) in 15% yield, together with the modified biaryl compounds **8** and **9** in 14% combined yield.

The phenolic products generated in these reactions are not stable under the oxidation conditions, consistent with observations noted in previous studies of lignin conversion to monomers under related conditions.^[^ ^28^, ^31^, ^36^, ^55^ ^]^ For example, monomeric phenols have been shown to undergo ring cleavage to afford low molecular weight (C_1_–C_6_) carboxylic acid products.^[^ ^31^, ^56^ ^]^ The monomer instability complicates batch reactions of this type because much of the reaction time involves heating the reactor to the desired temperature, limiting control over the reaction conditions. We anticipated that improved outcomes could be achieved by using a flow reactor that allows for more precise control of the reaction conditions.^[^ ^57^, ^58^, ^59^ ^]^ We have previously used a flow reactor that employs O_2_‐permeable poly(tetrafluoroethylene) (PTFE) tubing contained in a pressure vessel, permitting continuous delivery of O_2_ to a solution passing through the reactor (see schematic in Figure 2a).^[^ ^36^, ^60^ ^]^ Moving the oxidation to flow allows the mixture to be heated rapidly and the oxidation reaction to be run at much higher temperatures than in batch (up to 210 °C). The reaction mixture exits into a water‐jacketed stainless‐steel flow loop maintained at room temperature, rapidly cooling the solution and stopping conversion.

Each of the dimer models **1**–**4** was subjected to alkaline aerobic oxidation in flow (Figure 2c–f).^[^ ^61^ ^]^ As seen from the time course data for the oxidation of the model compounds in flow (Figure 2c–f), the substrates were quickly and completely converted within ∼30 s at these conditions. The highest monomer yield for the cleavage of the β‐1 (**1**), β‐5 (**2**), and β‐β (**3**) compounds afforded 64 mol%, 66 mol%, and 60 mol% yields of monomers, respectively. Aldehydes (vanillin and syringaldehyde) are the most prevalent products at the point of highest overall monomer yield, with the other major products consisting of aceto‐ and carboxylic acid‐substituted phenols. The biaryl model compound **4** contains a comparatively inert C_Ar_─C_Ar_ bond. Under the reaction conditions, this substrate yields a different set of aromatic products, including ortho‐substituted G‐type monomers arising from C─C cleavage, 5‐carboxyvanillic acid (**5**), 4‐hydroxy‐5‐methoxyisophthaldehyde (**6**), 5‐formyl‐2‐hydroxy‐3‐methoxybenzoic acid (**7**), and dimer products **8** and **9** (Figure 2f). The dimers arise from side chain oxidation of compound **4** without C_Ar_─C_Ar_ bond cleavage. With all compounds, longer reaction times led to degradation of the aromatic compounds, aligning with the batch reaction results. Collectively, the comparison of monomer yields obtained in the batch and flow reactions (Figure 2b) highlights the improved results accessible in the flow reactor.

### Analysis of Monomer Degradation under Oxidative Reaction Conditions

**Figure 3:**
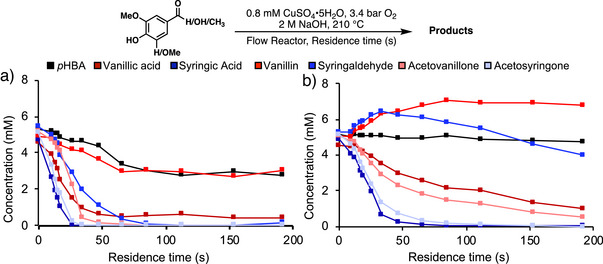
a) Conversion/degradation of individual aromatic monomers under flow‐based alkaline aerobic oxidation. b) Oxidation of combined (5 mM each) aromatic monomers.

To gain further insights into monomer degradation in these reactions, the individual guaiacyl (G)‐derived (vanillic acid, vanillin, acetovanillone) and syringyl (S)‐derived compounds (syringic acid, syringaldehyde, and acetosyringone) (5 mM each) were subjected to aerobic oxidation in flow. The time‐course data (Figure 3a) reveals that the S‐derived compounds (blue) degrade more rapidly than their G‐derived counterparts (red). *p*‐Hydroxybenzoic acid (*p*HBA, black data in Figure 3) is a component in poplar‐derived RCF oils, and it underwent degradation at a rate similar to that observed for vanillin.

When the same monomer components were evaluated as a mixture (5 mM each, 35 mM total monomers) (Figure 3b), the degradation rates were somewhat slower, likely reflecting a competition for oxygen among the different compounds. The concentration of *p*HBA is steady throughout the reaction of the mixed compounds, while the concentrations of vanillin and syringaldehyde increase slightly at the beginning of the reaction before they begin degrading. The increase in aldehyde monomers arises from the conversion of acetovanillone and acetosyringone into vanillin and syringaldehyde, respectively, during their degradation (Figure S3), which will be the focus of future investigation.

### Oxidation of Oligomers in RCF Lignin Oil Derived from Pine and Poplar Biomass

**Figure 4:**
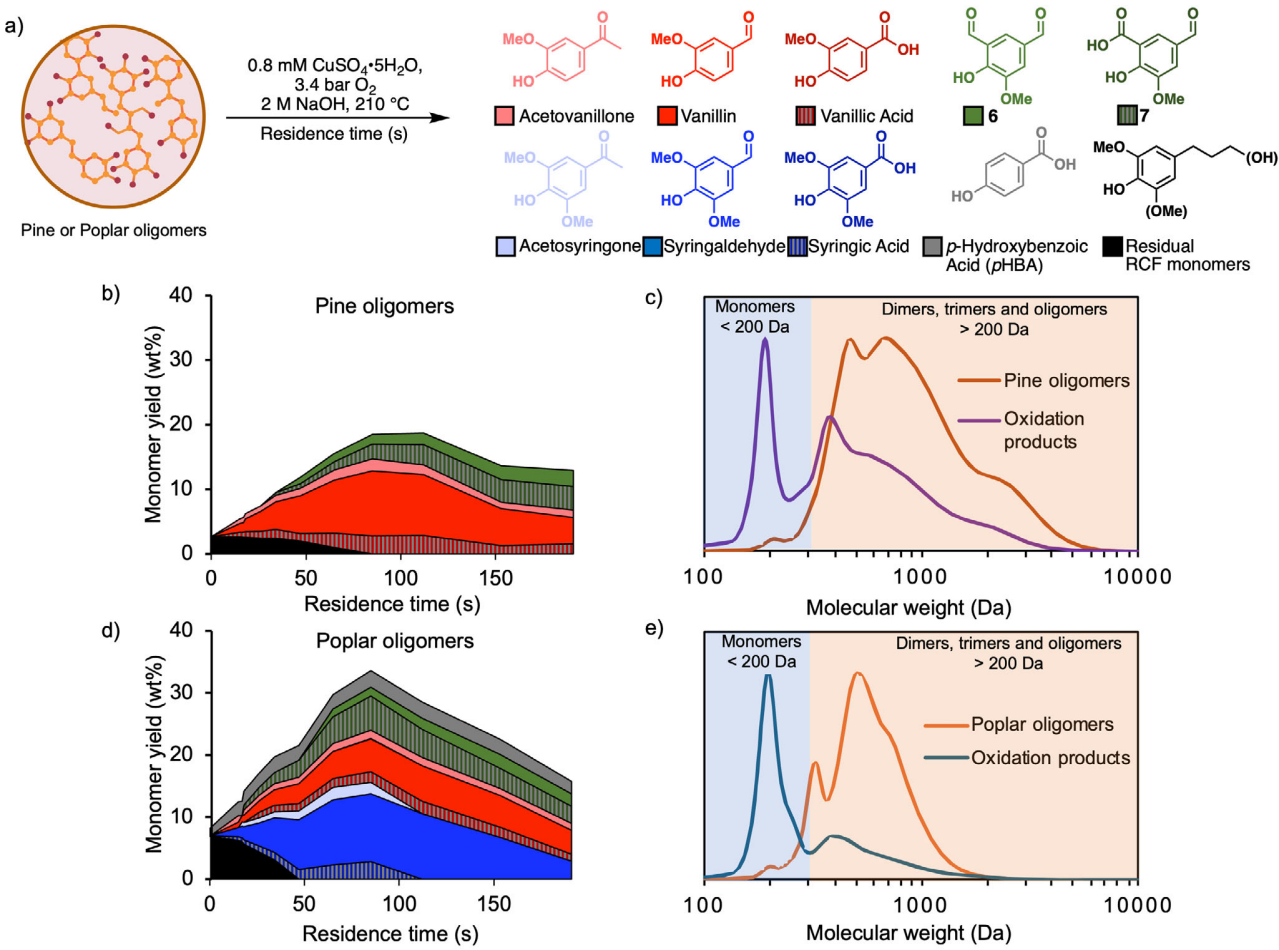
a) Oxidation of pine‐ and poplar‐derived RCF oligomers in the flow reactor. Time‐course profiles for the oxidation of pine (b) and poplar (d) oligomers. Gel permeation chromatogram of oligomers and the oxidation products after workup at maximum yield for c) pine and e) poplar RCF oligomers.

We then sought to assess the viability of flow‐based alkaline aerobic oxidation on mixtures of oligomers derived from the RCF process with pine and poplar biomass. RCF oil containing monomeric phenol compounds (e.g., propanolguaiacol, propanolsyringol) and C─C linked oligomers was generated using Ru/C‐catalyzed hydrogenolysis of extractive‐free biomass in methanol, as previously reported (see Section 3 of the Supporting Information).^[^ ^25^ ^]^ The resulting RCF product oils from pine and poplar contain 25 wt% and 48 wt% phenolic monomers, respectively, with the balance consisting of C─C‐linked oligomers. The methanol solvent and monomers were removed by vacuum distillation to obtain the RCF oligomer oils that were used as the feedstocks in this study. The oligomer‐rich residues had low, but detectable, monomer content: 2.6 wt% monomers in the pine and 6.9 wt% monomers in the poplar (evident at the origin of plots in Figure 4b,d).

To identify optimal deconstruction conditions, time‐course analysis of the oxidation of pine and poplar RCF oligomers was performed using the O_2_‐permeable membrane reactor (see Figures S4 and S5 for details). Initial conditions were based on our prior work on alkaline aerobic oxidation using the same O_2_‐permeable membrane reactor,^[^ ^36^ ^]^ and temperature, O_2_ pressure, copper concentration, NaOH concentration, and oligomer concentration were varied. These results showed that changes to O_2_ pressure primarily influence reaction rate while changes to Cu and NaOH concentration have little effect on the yields of aromatic monomers beyond a threshold of 0.8 mM and 2 M, respectively. The largest effect arose from increasing the temperature, and the best yields were obtained at a temperature of 210 °C. Treatment of the oligomers from pine afforded five G‐type monomers: vanillic acid, vanillin, acetovanillone, dialdehyde **6,** and 5‐carboxyvanillin **7**, as quantified by UPLC (Figures 4b and S7). A maximum monomer yield of 19 wt% was achieved at 85 s residence time for pine oligomers (Figure 4b). RCF oligomers derived from poplar, subjected to the same conditions, led to the same G‐type monomers in addition to S‐type monomers (syringic acid, syringaldehyde, and acetosyringone) and *p*HBA. The *p*HBA is not an oxidation product as it was present in the original oligomer sample, esterified to the oligomers, and is readily released via hydrolysis under the basic reaction conditions.^[^ ^62^ ^]^ A maximum monomer yield of 34 wt% was again obtained at 85 s residence time (Figure 4d). Longer reaction times led to monomer degradation and lower monomer yields in both cases, matching the behavior of the model compounds. The higher reactivity of S aromatic units compared to G aromatic units led to more rapid monomer degradation in the hardwood samples relative to softwood samples.^[^ ^55^ ^]^

For gel permeation chromatography (GPC) and biological funneling experiments, continuous, gram‐scale conversion of RCF oligomers was conducted by feeding an aqueous NaOH solution of the oligomers through the flow reactor with a constant 85 s residence time. The resulting product solution was acidified to pH 2 to ensure protonation of the carboxylic acids and then extracted with ethyl acetate to remove the organic materials from the high‐salt solution. The oxidation product mixtures obtained following solvent removal were enriched in phenolic monomers: 18 wt% monomers from the reaction of pine RCF oligomers and 42 wt% monomers from the poplar RCF oligomers. GPC of these samples revealed a significant decrease in oligomeric (>200 Da) content and large peaks corresponding to low molecular‐weight compounds, consistent with the expected C─C cleavage in the RCF oligomers (Figure 4c,e). A lower abundance of high molecular weight structures is evident in the poplar oxidation products relative to the pine‐derived products. This feature likely reflects the tendency of the less sterically‐hindered G subunits in pine lignin to undergo condensation, either during biosynthesis^[^ ^63^ ^]^ or in the lignin extraction process,^[^ ^13^, ^14^ ^]^ forming unreactive C_Ar_─C_Ar_ bonds that are less amenable to cleavage under these oxidation conditions.^[^ ^64^ ^]^ Previous work, for example, has shown that the 5–5 C_Ar_─C_Ar_ fragment is enriched in RCF pine oil (10.9 wt%).^[^ ^13^ ^]^

### Comparison of Oxidative Strategies for Lignin and RCF‐Oligomer Deconstruction to Monomers Under Alkaline Aerobic Oxidation Conditions

**Figure 5:**
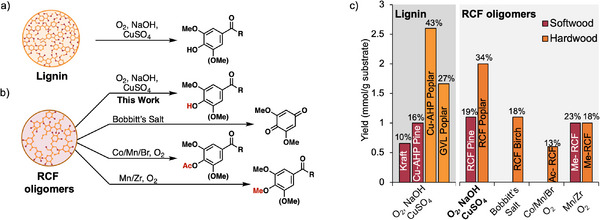
a) Alkaline aerobic oxidation of lignin. b) Methods for oxidative C─C cleavage in RCF oligomers. c) Yields for flow based alkaline aerobic lignin oxidation (left)^[^ ^26^ ^]^ compared to this work (**bold**) and literature reported methods for oxidative C─C bond cleavage in RCF lignin oligomers (right).^[^ ^16^, ^17^, ^18^ ^]^ See text for details.

The above results, showing that alkaline aerobic oxidation conditions cleave C─C bonds in lignin‐derived oligomers to access monomers, provide a basis for comparison with two other sets of results: (1) alkaline aerobic oxidation of lignin obtained from biomass pretreatment, and (2) other oxidation methods used to convert RCF oligomers into monomers (Figure 5).

Pretreatment lignin was recently subjected to identical alkaline aerobic conditions in flow to access mixtures of monomers and oligomers.^[^ ^36^ ^]^ Representative results, illustrated in Figure 5c (*left*), come from oxidative deconstruction of several lignin sources: a commercial source of kraft pine lignin, derived from industrial pulp and paper manufacturing, pine and poplar lignin obtained from a copper/hydrogen‐peroxide‐based pretreatment method that retains significant β‐aryl ether bonds in the extracted lignin (Cu‐AHP),^[^ ^65^, ^66^ ^]^ and a mildly acidic organosolv‐type pretreatment process that uses γ‐valerolactone (GVL) as a process solvent.^[^ ^67^ ^]^ The monomer yields obtained from these lignin sources mirror results obtained with the RCF oligomers, with the softwood pine‐derived material affording lower yields of monomers than obtained from the poplar‐derived materials. A notable outcome, however, is that the monomer yields obtained from RCF oligomers are competitive with those from the best pretreatment lignin sources despite the absence of readily cleavable β‐aryl ether bonds. Specifically, the 19 wt% monomers from RCF pine oligomers exceeds the 10 and 16 wt% monomers obtained from kraft and Cu‐AHP pine lignin, and the 34 wt% monomers from RCF poplar oligomers falls between the yields of monomers obtained from GVL and Cu‐AHP poplar lignin (27 and 43 wt%, respectively).

The data also may be compared on the basis of mmol of monomeric products with respect to the mass of original substrate (lignin or RCF oil). This metric is better for comparison of different RCF oligomer oxidation methods outlined in the Introduction.^[^ ^24^, ^25^, ^26^ ^]^ These different methods yield products with varied molecular weights, owing to the loss of side chains in the production of dimethylbenzoquinone with stoichiometric Bobbitt's salt^[^ ^24^ ^]^ or from the presence of phenol‐protecting groups (acetyl,^[^ ^25^ ^]^ methyl^[^ ^26^ ^]^) in the catalytic autoxidation reactions. In addition, the latter autoxidation reactions primarily generate carboxylic acids, which have a higher molecular weight than aldehydes, which are the major products of the present alkaline aerobic oxidation method. Despite these complications, the comparison of the different methods in Figure 5c (*right*) highlights the effectiveness of the present conditions. In addition to achieving higher mmol g^−1^ product yields relative to the other methods, the alkaline aerobic oxidation conditions use catalytic amounts of inexpensive Cu salts and require no protection of the phenols prior to the reaction.

Ultimately, the sequence of RCF treatment of biomass followed by oxidative treatment of the RCF oligomers provides two routes to form aromatic monomers from lignin, significantly enhancing the overall yield of monomeric products. In the present study, this sequence affords a total aromatic monomer yield of 33 wt% (1.9 mmol g^−1^) from pine and 61 wt% (3.5 mmol g^−1^) from poplar with respect to the mass of corresponding RCF oils (see Tables S6 and S7 and the associated text in the Supporting Information for yield tabulation).

### Biological Funneling of Product Mixture to PDC and Muconate

**Figure 6:**
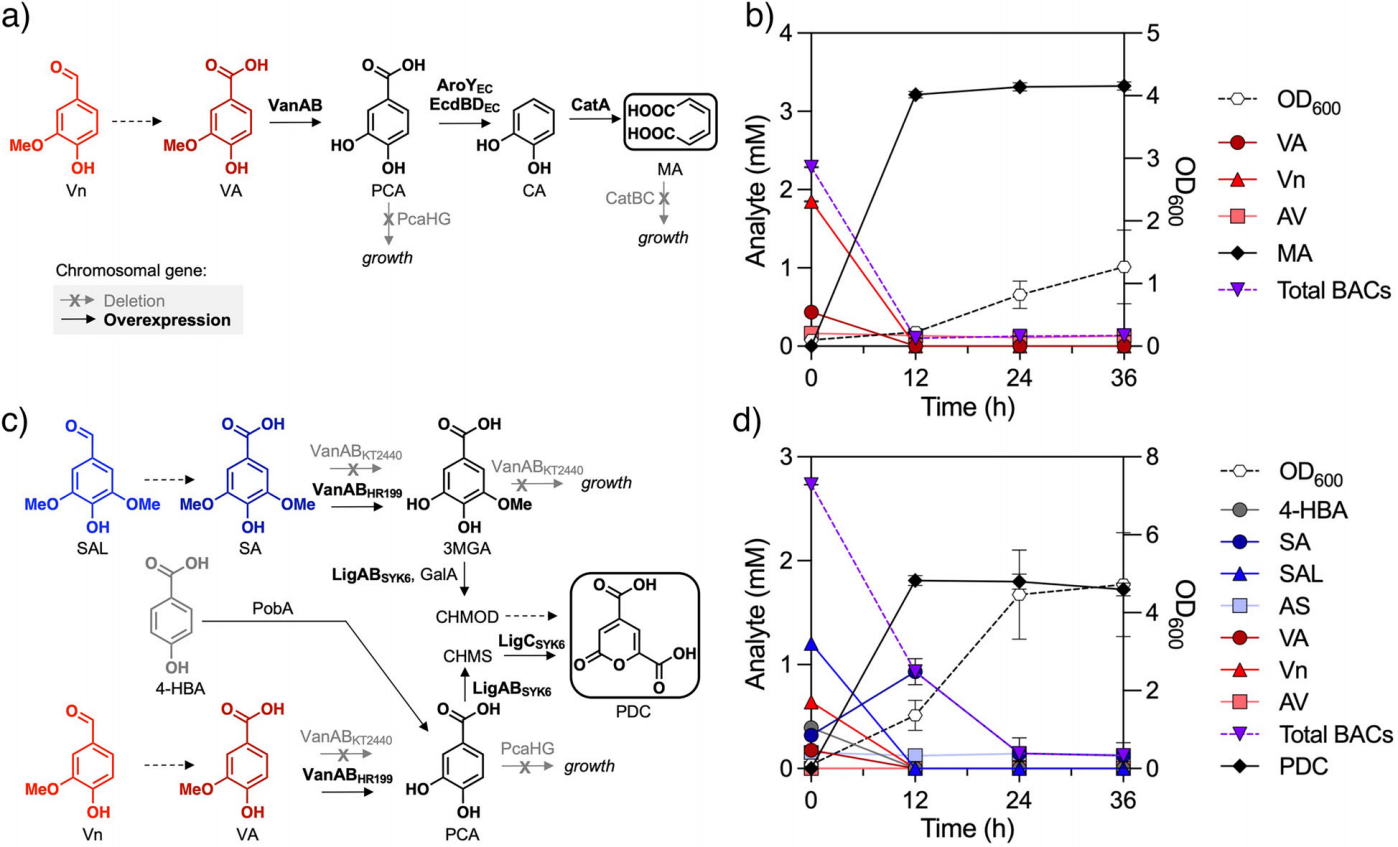
Bioconversion of lignin oil to (a, b) muconate or (c, d) PDC. Metabolic pathways for the conversion of a) vanillin (Vn) and vanillate (VA) to muconate (MA) in engineered *P. putida* KT2440 strain, CJ781, and c) Vn, VA, 4‐hydroxybenzoate (4‐HBA), syringaldehyde (SAL), syringate (SA) to 2‐pyrone‐4,6‐dicarboxylic acid (PDC) in the engineered *P. putida* KT2440 strain, AW045. Deletion, and overexpression of select genes are denoted in gray and bold, respectively. b) CJ781 and d) AW045 were cultivated in shake flasks at 30 °C/225 rpm in M9 minimal medium supplemented with b) 10 mM glucose and pine‐derived deconstructed lignin oil or (d) 40 mM glucose and poplar‐derived deconstructed lignin oil. CJ781 cultivations were fed to 10 mM glucose at 12, 24, and 48 h. AW045 cultivations were fed to 20 mM glucose every 24 h. Chemical abbreviations: VA, vanillate; Vn, vanillin; AV, acetovanillone; MA, muconate; 4‐HBA, 4‐hydroxybenzoate; SA, syringate; SAL, syringaldehyde; AS, acetosyringone; 2‐pyrone‐4,6‐dicarboxylic acid, PDC; BACs, bioavailable compounds; OD_600_, optical density, measured as absorbance at 600 nm. Data points and error bars represent the mean and standard deviation, respectively, of n ≥ 3 biological replicate.

We next sought to convert the mixture of lignin‐derived oxidized aromatic monomers into a single bioproduct using engineered *P. putida* KT2440 (hereafter KT2440; Figure 6). KT2440 is a soil‐dwelling bacterium with high salt and aromatic tolerance and a versatile metabolism.^[^ ^68^, ^69^, ^70^ ^]^ Here, we leveraged previously engineered strains to convert pine‐derived lignin oils into *cis,cis*‐muconic acid (muconate),^[^ ^46^ ^]^ a precursor for adipic acid or other performance‐advantaged bioproducts, and poplar‐derived lignin oils into 2‐pyrone‐4,6‐dicarboxylic acid (PDC).^[^ ^47^ ^]^ KT2440 has previously been engineered to convert lignin‐derived aromatics to muconate via heterologous expression of the *aroY* gene encoding a protocatechuate decarboxylase, overexpression of the *catA* gene encoding the native catechol dioxygenase, and deletion of *pcaHG* and *catBC* in strain CJ781^[^ ^46^ ^]^ (Table S8, Figure 6a). However, the strain cannot metabolize acetovanillone or compounds **5**–**7** which are minor products in the oxidative mixtures. Here, we cultivated CJ781 in pine‐derived lignin oil and observed simultaneous substrate conversion with muconate production at 127 ± 11% molar yields. Substrate utilization and production dynamics were similar to a mock mixture of vanillin and vanillate (Figure S10a) suggesting microbial toxicity effects from the oxidized lignin effluent were minimal. Notably, muconate yields on pine‐derived oils exceed 100%, suggesting additional unknown compounds (not quantified) in the oil were utilized as has been previously reported.^[^ ^71^, ^72^, ^73^ ^]^

For poplar‐derived lignin oils, PDC production was pursued as no metabolic pathways are known from S‐type compounds to muconate. KT2440 was previously engineered to simultaneously convert S‐, G‐, and H‐type aromatic monomers to PDC in strain AW045 (Table S8, Figure 6c).^[^ ^47^ ^]^ Similar to CJ781, the strain cannot metabolize acetovanillone or acetosyringone, nor compounds **5**–**7**. When cultivated in the presence of poplar‐derived lignin oil, AW045 simultaneously converted syringaldehyde, syringate, vanillin, vanillate, and 4‐hydroxybenzoate to PDC at 57 ± 2 mol% yields (Figure 6d). Similar aromatic acid utilization but higher PDC yields were achieved on a mock mixture of aromatic monomers (Figure S10b), suggesting a component of the poplar‐derived lignin oil may affect strain performance. Notably, PDC yields from a mock mixture are less than 100 mol%, suggesting carbon loss through uncharacterized enzymatic activity on PDC or one of the intermediates (Figure S10).

## Conclusion

The results outlined above demonstrate the effectiveness of Cu‐catalyzed alkaline aerobic oxidation conditions to cleave C─C bonds and convert RCF lignin‐derived oligomers from pine and poplar biomass into monomers. The catalytic oxidation conditions are compatible with phenols, allowing the process to operate directly with RCF oligomers, without requiring phenol‐protecting groups. The use of a flow reactor enables access to substantially higher monomer yields compared to batch oxidation conditions, leveraging the improved control over residence time, temperature, and oxygen delivery. More broadly, the sequence of reductive and oxidative process steps, consisting of biomass RCF and oxidative deconstruction of RCF oligomers, enhances the available aromatic monomer yields accessible from lignin.

The oxidative method yields an array of bioavailable aromatic monomers, including vanillic acid, vanillin, acetovanillone, syringic acid, syringaldehyde, and acetosyringone, which are efficiently funneled into *cis*,*cis*‐muconic acid and 2‐pyrone‐4,6‐dicarboxylic acid using engineered *Pseudomonas putida* KT2440 strains. This bioconversion process reduces purification requirements by eliminating the need for additional isolation and purification of individual monomers, highlighting opportunities enabled by integration of chemical and biological approaches for lignin valorization. These results establish an important foundation for future work, including technoeconomic analysis that considers integration of the different process steps, catalyst recycling, and product purification, to support large‐scale viability.

## Conflict of Interests

The authors declare no conflict of interest.

## Supporting information



Supporting Information

## Data Availability

The data that support the findings of this study are available in the Supporting Information of this article.
