# Supplementary material for: Cu‐Catalyzed Aerobic Oxidative C─C Cleavage in Lignin‐Derived Oligomers and Biological Funneling of the Monomeric Products
Source: Angew Chem Int Ed Engl. 2025 Nov 17;65(2):e15588. doi: 10.1002/anie.202515588 (PMC12790356; doi:10.1002/anie.202515588)
Supplement: Supplementary file 1 — Supporting Information [file ANIE-65-e15588-s001.pdf]

Supporting Information

Cu-Catalyzed Aerobic Oxidative C–C Cleavage in Lignin-Derived Oligomers and Biological Funneling of the Monomeric Products

Surajudeen Omolabake,[1,2] Dillon T. Hofsommer,[1,2] Kathryn M. Mains,[3,4] Chad T. Palumbo,[3] Davide Rigo,[3,4] Allison Z. Werner,[3,4] Gregg T. Beckham,[3,4,] and Shannon S. Stahl[1,2,*]*

[1]*Department of Chemistry, University of Wisconsin-Madison, 1101 University Avenue Madison, WI, 53706, United States*

[2]*Wisconsin Energy Institute, University of Wisconsin–Madison, Madison, Wisconsin 53726, United States*

[3]*Renewable Resources and Enabling Sciences Center, National Renewable Energy Laboratory, Golden, Colorado 80401, United States*

[4]*Center for Bioenergy Innovation, Oak Ridge National Laboratory, Oak Ridge, Tennessee, 37830, United States*

*Corresponding Author: stahl@chem.wisc.edu, gregg.beckham@nrel.gov

Table of Contents:

1. **General Experimental Considerations** **S2**
2. **Synthesis of Model Compounds** **S2**
3. **Preparation of RCF Substrates** **S6**
4. **Oxidation of Model Compounds in Batch** **S6**
5. **Oxidation of Model Compounds and RCF Substrates by O_2-Permeable Membrane Flow Reactor** **S8**
6. **Biological Conversion** **S16**
7. **Analytical Methods** **S18**
8. **NMR and Mass Spectra** **S25**
9. **References** **S31**

1. General Experimental Considerations

All commercial reagents were purchased and used as received. Reagent grade pentane, reagent grade ethyl acetate, reagent grade methanol, reagent grade dichloromethane, 1,4-dimethoxybenzene, sodium hydroxide, vanillin, syringaldehyde, vanillic acid, *p*-hydroxybenzoic acid and syringic acid were purchased from Sigma-Aldrich. $CuSO_4$, UPLC grade methanol, and UPLC grade ethyl acetate were purchased from Fischer. 4-Hydroxy-5-methoxyisophthaldehyde, 3-formyl-4-hydroxy-5-methoxybenzoic acid, 5-carboxyvanillic acid, and 5-formyl-2-hydroxy-3-methoxybenzoic acid were purchased from Ambeed.

Flow lignin depolymerization reactions were performed using a custom-built flow reactor[1] constructed from a 300 mL stainless steel Parr vessel, modified with two nominal 1/4 inch NPT fittings tapped into the bottom of the vessel and equipped with a burst disc rated to 138 bar. The reaction temperature was precisely regulated by a Parr 4838 temperature controller with a K-Type thermocouple. A Hitachi L6200 Intelligent pump was utilized, fitted with PTFE tubing (1.58 mm OD × 0.8 mm ID × 5 ft) obtained from Supelco. The tube pressure was managed via a dome valve back-pressure regulator, adjustable using a specified nitrogen pressure.

Ultra-High Performance Liquid Chromatography (UHPLC) analysis was performed using a Waters Acquity Class H QSM Plus UPLC system, equipped with a BEH C18 1.7 µm, 2.1 x 50 mm column, heated to 40 °C. Data acquisition was done using Empower software, with calibration curves and sample traces obtained via a photodiode array monitoring elution at 280 nm ± 0 nm bandwidth.

High resolution mass spectrometry (HRMS) was performed using a Thermo Q Exactive Plus™ mass spectrometer.

Safety Note – When conducting reactions at temperatures significantly above the solvent's boiling point, exercise caution. Ensure the operating pressure remains well within the reactor vessel's safety limits, which should have a fail-safe burst disk. Always use a blast shield and heat-resistant gloves when handling a pressurized, heated vessel.

2. Synthesis of Model Compounds

2.1 Synthesis of Dendrophenol (β-1 dimer) (**1**). Dendrophenol was synthesized according to literature procedure with slight modifications.[2]

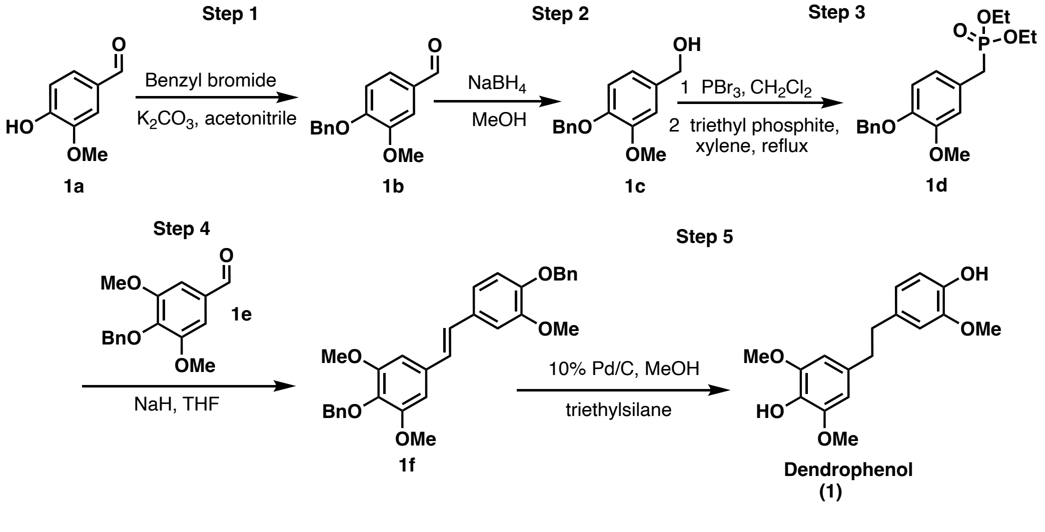

Step 1: To a stirred solution of vanillin **1a** (10 mmol, 1.5 g) in acetonitrile (75 mL) was added K_2CO_3 (12 mmol, 1.7 g) under nitrogen atmosphere and cooled to 0 °C using an ice-water bath. Benzyl bromide (12 mmol, 2.1 g) was added, and the mixture was stirred overnight at room temperature. The reaction mixture was filtered to remove the base, and product was concentrated using a rotary evaporator to afford the white solid **1b** (2.4 g, 98% yield) which was used in the next step without further purification. The NMR data is consistent with the previously reported values.

Step 2: To a stirred solution of protected vanillin **1b** (2.5 mmol, 0.6 g) in MeOH (10 mL) at 0 °C was added sodium borohydride (3.0 mmol, 0.1 g) portion-wise. The reaction mixture was stirred for 30 minutes at room temperature. The excess sodium borohydride was quenched with water (2 mL), stirred for 15 min, and the solvent was removed using a rotary evaporator. The crude mixture was redissolved in EtOAc (30 mL) and H_2O (20 mL). The aqueous layer was then extracted with EtOAc (3x30 mL). The organic phase from each extraction was combined and washed with brine (30 mL) and dried over Na_2SO_4 before the organic solvent was removed under reduced pressure. The crude was purified by column chromatography with pentane/EtOAc 50:50 to afford the target product **1c** (0.6 g, 95% yield). The NMR data is consistent with the previously reported values.

Step 3: To a stirred solution of **1c** from step 2 above (2.4 mmol, 0.6 g) in CH_2Cl_2 (6 mL) at 0 °C was added phosphorus tribromide (1.0 mmol, 0.3 g) and allowed to stir for 1 hour. The excess phosphorus tribromide was quenched with saturated $NaHCO_3$ solution and extracted with CH_2Cl_2 (3 x 30 mL). The organic layer was recovered, combined and washed with brine (30 mL), and dried over Na_2SO_4 before the crude solution was concentrated. The crude was redissolved in xylene (10 mL) and triethyl phosphite (3.0 mmol, 0.5 g) was added and the resulting mixture was refluxed overnight. The product mixture was concentrated using a rotary evaporator and the crude product was recovered, which was purified using Combiflash™ using pentane/EtOAc 50:50 to the target compound **1d** (0.8 g, 88% yield). The NMR data is consistent with the previously reported values.

Step 4: To a stirred solution of sodium hydride (2.4 mmol, 0.06 g) in anhydrous THF (5 mL) at 0℃ was the Wittig reagent **1d** from step 3 above (2.0 mmol, 0.7 g) in anhydrous THF (5 mL) dropwise under nitrogen atmosphere. The solution was stirred for 30 minutes after which the protected syringaldehyde (1.6 mmol, 0.4 g) in anhydrous THF (5 mL) was added. The mixture was stirred overnight at room temperature. To work up the reaction, ice cold water (5 mL) was added and extracted with EtOAc (3 x 30 mL). The combined organic layer was washed with brine (30 mL), dried over Na_2SO_4 and concentrated using a rotary evaporator. The crude mixture was further purified using Combiflash™ pentane/EtOAc 50:50 to give target compound **1e** (0.6 g, 78% yield). The NMR data is consistent with the previously reported values.

Step 5: To a stirred solution of **1e** (1.2 mmol, 0.6 g) in MeOH (10 mL) was added 10% Pd/C (0.04 g). Triethylsilane (18 mmol, 2.1 g) was then added, and the mixture was allowed to stir at room temperature with occasional venting. After 25 minutes, the mixture filtered through a celite pad and washed with EtOAc (40 mL) and concentrated using a rotary evaporator. The crude was purified using a Combiflash™ automated system with pentane/EtOAc 1:1 to give the β-1 dimer model dendrophenol **1** which was dried overnight under high vacuum (0.3 g, 90% yield). ^{1}H NMR (500 MHz, $CDCl_3$): δ 6.83 (d, J = 10 Hz, 1H) 6.67 (dd, J = 10 Hz, 2.0 Hz, 1H), 6.61 (d, J = 2 Hz, 1H), 6.36 (s, 2H), 5.50 (s, 1H), 5.40 (s, 1H), 3.84 (d, J = 4 Hz, 9H), 2.81 (s, 4H). ^{13}C NMR (126 MHz, DMSO-d_6) δ 146.90, 146.32, 143.83, 133.76, 132.99, 132.85, 121.16, 114.25, 111.29, 105.16, 56.34, 55.97, 38.63, 38.08. HRMS (ESI) m/z: $[M-H]^-$ calc. for $C_{17}H_{20}O_5$ 303.1238; found, 303.1238

2.2 Synthesis of 2-(1-(4-hydroxy-3-methoxyphenyl)propan-2-yl)-6-methoxy-4-(prop-1-en-1-yl)phenol (β-5 dimer) (2).

The β-5 dimer was synthesized in four-steps starting with coniferyl alcohol according to the literature procedures.[3,4]

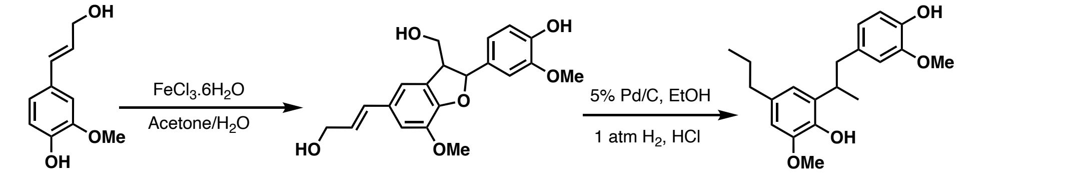

2a **2b** **2-(1-(4-hydroxy-3-methoxyphenyl)propan-2-yl)-6-methoxy-4-(prop-1-en-1-yl)phenol (2)**

Step 1: To a stirred solution of coniferyl alcohol **2a** (22.2 mmol, 4.0 g) in acetone (100 mL) and water (450 mL) was added a solution of $FeCl_3$•$6H_2O$ (44.8 mmol, 12.2 g) in water (50 mL). The mixture was stirred for 1 hour and extracted using EtOAc (3 x 200 mL). The organic layer was recovered and combined and washed with sodium ascorbate (0.1 M, 200 mL), brine (300 mL), and dried with Na_2SO_4. The organic solvent was removed using a rotary evaporator to give the crude compound which was further purified using a Combiflash™ automated system using pentane/acetone to afford dehydrodiconiferyl alcohol **2b** (1.0 g, 25% yield). The NMR data is consistent with the previously reported values.

Step 2: To a stirred solution of dehydrodiconiferyl alcohol (0.7 mmol, 0.2 g) in EtOH (10 mL) at 0 °C was added 5% Pd/C (50 mg) followed by 12 M HCl (1 mL). The reaction mixture was stirred under H_2 atmosphere overnight at room temperature. Monitoring by thin layer chromatography indicated all the starting materials was converted. The catalyst was filtered off using a polyamide membrane (0.2 μm), and the product was recovered by evaporation of the solvent using a rotary evaporator. The crude product was purified using a Combiflash™ automated system using pentane/EtOAc 50:50 to give the β-5 dimer model (0.2 g, 85% yield). ^{1}H NMR (500 MHz, $CDCl_3$) δ 6.79 (d, J = 8.0 Hz, 1H), 6.66 (dd, J = 8.0, 1.9 Hz, 1H), 6.62 (d, J = 1.9 Hz, 1H), 6.59 (d, J = 1.9 Hz, 1H), 6.55 (d, J = 1.9 Hz, 1H), 5.57 (s, 1H), 5.44 (s, 1H), 3.87 (s, 3H), 3.81 (s, 3H), 3.37 (dt, J = 8.6, 6.4 Hz, 1H), 2.95 (dd, J = 13.4, 5.9 Hz, 1H), 2.63 (dd, J = 13.4, 8.7 Hz, 1H), 2.53 – 2.43 (m, 2H), 1.59 (q, J = 7.5 Hz, 3H), 1.18 (d, J = 7.0 Hz, 3H), 0.93 (t, J = 7.3 Hz, 3H). ^{13}C NMR (126 MHz, $CDCl_3$) δ 146.04, 145.97, 143.51, 140.79, 133.70, 133.31, 132.08, 121.89, 119.12, 113.79, 111.68, 108.36, 55.98, 55.79, 42.81, 38.08, 34.59, 25.05, 19.36, 13.90. HRMS (ESI) m/z: $[M-H]^-$ calc. for $C_{20}H_{26}O_4$ 329.1758; found, 329.1758.

2.3 Synthesis of 5,5′-dimethoxysecoisolariciresinol (β-β dimer) (3)

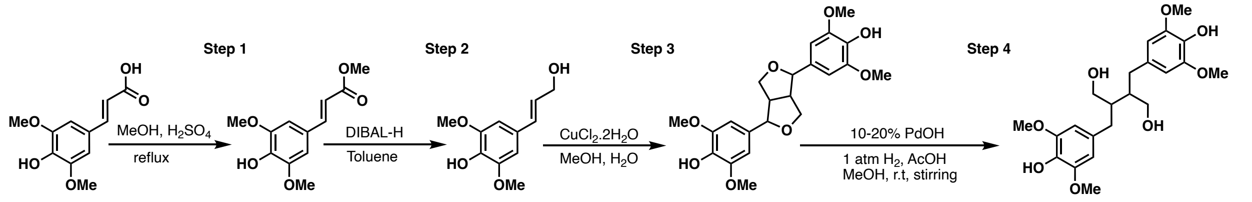

Sinapic acid 3a **Methyl sinapate 3b** **Sinapyl alcohol 3c** **Syringaresinol 3d** **5,5′-dimethoxysecoisolariciresinol (3)**

5,5′-dimethoxysecoisolariciresinol **3** was synthesized in a four-step procedure following literature procedures:

Step 1: Methyl sinapate **3b** was prepared following a slightly modified literature procedure.[3] Sinapic acid **3a** from Sigma-Aldrich (8.4 g, 37.4 mmol) was refluxed overnight in methanol (130 mL) in presence of 1 mL 72% H_2SO_4. The reaction mixture was cooled down to room temperature and the solvents were removed under reduced pressure using a rotary evaporator. The obtained solid was rinsed using cold methanol to give methyl sinapate as a white crystalline solid **3b** (8.0 g, 90% yield). The NMR data is consistent with the previously reported values.[3]

Step 2: To a solution of methyl sinapate **3b** (3.0 g, 12.6 mmol) in toluene (120 mL) was added DIBAL-H (50 mL of 1.0 M solution from Sigma-Aldrich) slowly via cannular transfer. The solution was stirred under nitrogen gas and monitored by TLC. After 2 hours of stirring, the reaction was worked up by quenching with 10 mL ethanol followed by product extraction using EtOAc three times (100 mL). The organic layer was washed with NH_4Cl solution (100 mL) and brine (100 mL) and dried with $MgSO_4$. The solvent was removed under pressure to give an off-white solid **3c** (2.6 g, 100% yield). The NMR data is consistent with the previously reported values.[5]

Step 3: Syringaresinol **3d** was prepared following a literature procedure with slight modifications.[3] To a solution of sinapyl alcohol (2.6 g, 12.4 mmol) in MeOH/water (20 mL/450 mL) was added $CuCl_2 \cdot 2H_2O$ (5.3 g, 31 mmol). The mixture was allowed to stir at room temperature for 24 hours. The product was extracted with 150 mL ethyl acetate four times. The organic layer was then washed with brine (100 mL) and dried with Na_2SO_4. The crude product is recovered by rotary evaporation and purified using a Combiflash™ system using pentane-ethyl acetate solvent with gradient elution running ethyl acetate from 0 to 90%. The collected fractions of the dissolved product were dried in vacuo to afford syringaresinol **3d** (1.3 g, 50% yield). The NMR data is consistent with the previously reported values.

Step 4: 5,5′-dimethoxysecoisolariciresinol **3** was prepared following a literature procedure.[6] To a stirred solution of syringaresinol (200 mg, 0.480 mmol) in MeOH (20 mL) and AcOH (1mL), was added 10-20% palladium hydroxide on carbon (from Aldrich, 100 mg). The mixture was stirred under 1 atm H_2 gas. After 3 hours of stirring, TLC analysis of the reaction mixture showed that the starting material was used up. The reaction mixture was then filtered, and the catalyst was washed with MeOH. The filtrate and washings were combined and evaporated to dryness in vacuo. The crude mixture was purified using a Combiflash™ system (pentane/EtOAc) to give the β-β dimer model 5,5′-dimethoxysecoisolariciresinol **3** (102.8 mg, 51% yield) as white crystals. ^{1}H NMR (500 MHz, DMSO): δ 8.03 (s, 2H), 6.33 (s, 4H), 4.55 (t, J = 5 Hz, 2H), 3.67 (s, 12H), 3.45-3.42 (m, 2H), 3.37-3.33 (m, 2H), 2.55-2.47 (m, 4H), 1.84-1.81 (m, 2H). ^{13}C NMR (126 MHz, DMSO) δ 147.69, 133.30, 131.43, 106.22, 60.30, 55.82, 42.40, 34.56. HRMS (ESI) m/z: $[M-H]^-$ calc. for $C_{22}H_{30}O_8$ 421.1868; found, 421.1868.

2.4 Synthesis of 3,3'-dimethoxy-5,5'-dipropyl-[1,1'-biphenyl]-2,2'-diol (5-5 dimer) (4).

The 5-5 model dimer was synthesized following a literature procedure.[7]

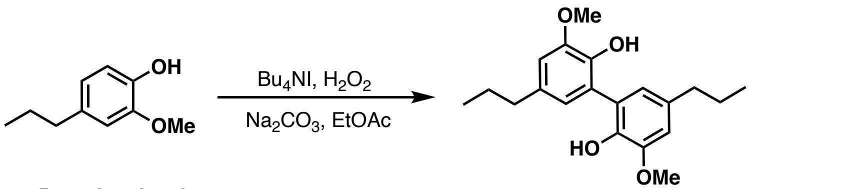

1-Propylguaiacol
4a

3,3'-dimethoxy-5,5'-dipropyl-[1,1'-biphenyl]-2,2'-diol
4

To a stirred solution of propyl guaiacol **4a** (Sigma-Aldrich, 1.0 mmol, 0.2 g) and Bu_4NI (0.1 mmol, 0.04 g) in EtOAc (5 mL) were added Na_2CO_3 (1.0 mmol, 0.1 g) and 30% H_2O_2 solution (1.2 mmol, 0.12 mL). The mixture was stirred for 8 hours at room temperature. The reaction mixture was poured into $NaHSO_3$ solution to quench the excess H_2O_2 (5 mL). A saturated solution of NH_4Cl (10 mL) was added to the mixture which

was then extracted with EtOAc (3 x 10 mL). The organic layer was recovered and combined and washed with brine (30 mL) and dried with Na_2SO_4. The organic solvent was removed using a rotary evaporator to give the crude compound which was further purified using a Combiflash™ automated system using pentane/EtOAc 50:50 to afford 5-5 model compound **4** (0.16 g, 100% yield). ^{1}H NMR (500 MHz, DMSO-*d*$_6$): δ 8.14 (s, 2H) 6.74 (d, *J* = 2 Hz, 2H), 6.53 (d, *J* = 2 Hz, 2H), 3.80 (s, 6H), 2.47 (t, *J* = 8 Hz, 4H), 1.57 (s, *J* = 8 Hz, 4H), 0.90 (t, *J* = 8 Hz, 6H). ^{13}C NMR (126 MHz, DMSO-*d*$_6$) δ 147.56, 141.42, 132.18, 125.85, 122.80, 110.68, 55.82, 37.11, 24.46, 13.83. HRMS (ESI) m/z: [M-H]$^-$ calc. for $C_{20}H_{26}O_4$ 329.1758; found, 329.1758.

3. Preparation of RCF Substrates

Production of pine and poplar RCF oil: A 7.6 L Parr reactor was loaded with 5 wt% Ru/C (15 g), nanopure DI water (15 g, to wet the catalyst), extractives-free pine or poplar (300 g), and methanol (3 L), and the reactor was subsequently sealed. A leak test of the reactor was conducted at 117.2 bar with N_2, followed by 2 purge cycles with N_2 at 34.5 bar. The system was then pressurized with H_2 to 30 bar at room temperature. The reactor was heated at 225 °C for 3 h with mechanical stirring. Afterwards, the reactor was cooled to room temperature with chilled water through an internal loop. The reaction mixture was passed through a frit to separate the catalyst and remaining biomass, and the filtrate was collected using a peristaltic pump. Methanol was removed from the filtrate by rotary evaporation at 80 mbar with heating to 35 °C. The resulting oil was washed with H_2O (ca. 400 mL) and extracted with EtOAc (ca. 400 mL, then ca. 120 mL x 3). The combined organic fraction was dried over Na_2SO_4 and concentrated under vacuum to yield the isolated RCF oil as a brown oil (60.99 g for poplar) and (55.03 g for pine). The RCF oil from poplar contained 48 wt% of RCF monomers while the RCF oil from pine contained 25 wt% of RCF monomers.

Monomer-oligomer separation from Pine RCF oil: A 25 mL round-bottom flask was charged with pine RCF oil (5.0 g) and attached to a Kugelrohr distillation apparatus equipped with a collection flask submerged in a dry ice / acetone bath. The apparatus was heated to 250 °C at 10 mbar for 30 min affording an oligomer-enriched residue fraction (2.2 g, 44 wt%) along with a pale-yellow distillate (1.5 g, 30 wt%) collected in the chilled flask. The total mass recovery was 74 wt%. The oligomer-enriched fraction contained 1.87 wt% of 4-propanolguaiacol.

Monomer-oligomer separation from Poplar RCF oil: A 25 mL round-bottom flask was charged with poplar RCF oil (5 g) and attached to a Kugelrohr distillation apparatus equipped with a collection flask submerged in a dry ice / acetone bath. The apparatus was heated to 270 °C at 10 mbar for 30 min affording an oligomer-enriched residue fraction (2.1 g, 43 wt%) along with a pale-yellow distillate (2.5 g, 49 wt%) collected in the chilled flask. The total mass recovery was 92 wt%. The oligomer-enriched fraction contained 4.61 wt% 4-propanolsyringol, 0.37 wt% 4-propanolguaiacol, and 0.34 wt% of 4-propylsyringol.

4. Oxidation of Model Compounds in Batch

A general procedure for the oxidation of the dimeric model compounds in batch is described: To a 100 mm tall, 26 mm O.D., and 24 mm I.D. PTFE vial were added a 1.5 mm x 7.9 mm PTFE coated stir bar, model compound was weighed and added to give ~ 3 mM substrate concentration, 10 mL 2 M aqueous sodium hydroxide, and 3.3 mg $CuSO_4 \cdot 5H_2O$ (1.3 mM). The solution was stirred at room temperature until the compound dissolved. 115 mL of water was added to a 1 L, stainless steel Parr reactor as a heating medium. The Parr vessel was wrapped with a heating mantle, affixed to a stir plate, sealed with a lid bearing a pressure gauge and thermocouple, and protected with a blast shield. The stirring was turned on, and the reactor was pressurized to with O_2. The heating mantle and thermocouple were connected to a Parr 4838 Reaction Controller tuned to a 130 °C (for a reaction performed at 130 °C) set point and turned on to heat

the reactions. After 37 minutes, when the reaction reached 130 °C, the heating was turned off and the reactor was submerged in a bucket of ice. When the reaction temperature fell to below 40 °C, the pressure was released, and the reactor was opened. 0.5 mL of the solution was transferred to a UPLC vial, followed by 0.5 mL of 1,4-dimethoxybenzene internal standard. 100 µL of 37% HCl was added to the UPLC vial to adjust the pH to 2 before the solution was injected into the UPLC and products quantified using a calibration curve and the internal standard (detailed in **Section 7**). Initial optimization was completed using the β-β model dimer **3** (**Figure S1** and **Table S1**) and was carried out in the same manner except for varying oxygen pressure, catalyst concentration, or temperature. The optimal conditions above were used for compounds **1, 2,** and **4** to generate the yields shown in **Figure 2** and **Table S2**.

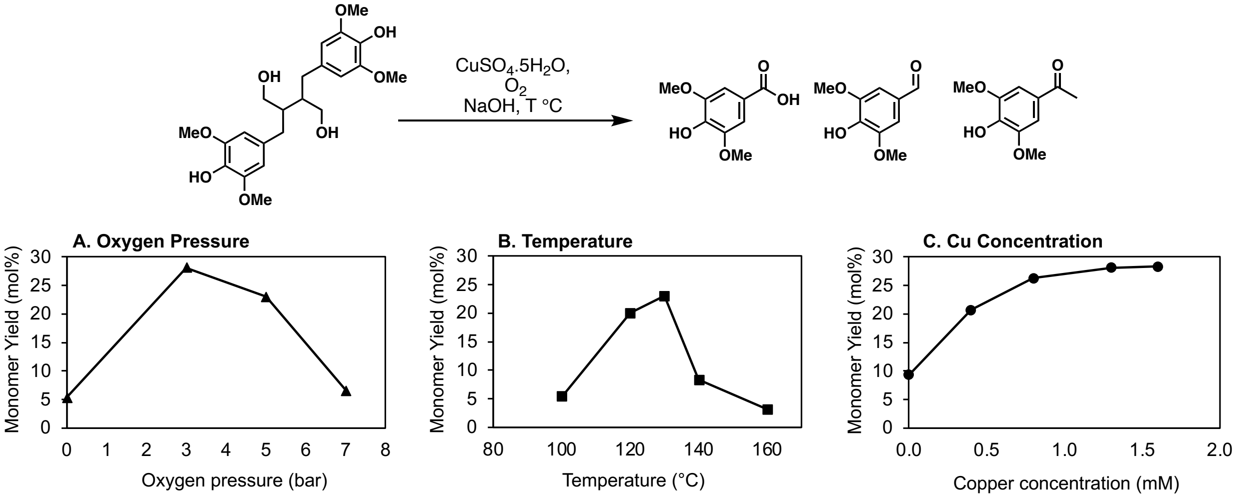

Figure S1. Optimization of oxygen pressure (A), temperature (B), and $CuSO_4$ concentration (C) for batch oxidations using the β-β model dimer. Reaction conditions: 3 mM β-β model substrate, 2 M NaOH (10 mL), 130 ºC, 1.3 mM [$CuSO_4$], 5 bar O_2, unless the parameter of interest was varied.

Table S1. Optimization of batch reaction conditions using β-β dimer **3**; temperature screen from 100 °C to 160 °C.

Time (min)	Temperature (°C)	Oxygen pressure (bars)	Catalyst conc. (mM)	Syringic acid	Syringaldehyde	Acetosyringone	Total Phenolics (mol%)
25	100	5	1.3	2.4	2.8	0.2	5.4
30	120	5	1.3	8.0	9.6	2.4	20.0
37	130	5	1.3	8.2	11.2	3.6	23.0
50	140	5	1.3	0.0	7.8	0.5	8.3
55	160	5	1.3	0.0	3.1	0.0	3.1
37	130	0	1.3	1.0	4.2	0.1	5.3
37	130	3	1.3	9.7	12.9	5.5	28.1
37	130	7	1.3	2.6	3.4	0.5	6.5
37	130	3	0.0	4.1	4.7	0.5	9.3
37	130	3	0.4	8.3	9.4	3.0	20.7
37	130	3	0.8	9.5	11.9	4.9	26.3
37	130	3	1.6	9.8	13.0	5.5	28.3

Reaction Conditions: 3 mM β-β model substrate, 10 mL of 2 M NaOH, and 3.3 mg $CuSO_4•5H_2O$.

5. Oxidation of Model Compounds and RCF Substrates by O_2-Permeable Membrane Flow Reactor

In a typical flow reaction, a 100 mL stock solution of 0.5 wt.% substrate (0.5 g) was prepared using 2 M NaOH. $CuSO_4 \cdot 5H_2O$ (0.020 g, 0.8 mM final concentration) was dissolved in 0.5 mL of Type 1 water and added to the stock solution while stirring. The mixture was then sonicated to ensure complete dissolution of the model compounds, pine oligomers, or poplar oligomers. The Parr vessel shell was pressurized to 3.4 bar with oxygen gas (unless otherwise noted) with a gas outlet rate of ca. 1 bubble per second. The back-pressure regulator was adjusted to 18 bar. Simultaneously, the flow reactor was preheated to the desired temperature (210 °C unless noted) and the HPLC pump was set to the required flow rate (0.24 mL/min to 3.00 mL/min), initially flowing deionized water to equilibrate the system. Once the reactor temperature stabilized, the pump was switched to draw from the stock solution. The reaction solution flows through the O_2-permeable tubing, where the oxidation reaction occurs. It then exits the pressurized reactor into water-cooled tubing, quenching the reaction, and continues through a back pressure regulator into a collection vessel. The stock solution was allowed to flow for at least three residence times before an aliquot was collected for analysis. To analyze a reaction time course, the flow rate was then adjusted, the reactor temperature was allowed to stabilize, and the solution was flowed for three more residence times to ensure steady operation before an aliquot was collected for analysis. The flowrate is controlled by an HPLC pump, and the volume of the reactor is calculated using the dimensions of the PTFE tube. For analysis by UPLC, a 0.5 mL aliquot was mixed with 0.5 mL of a 10 mM 1,4-dimethoxybenzene in methanol stock solution, used as an internal standard, and acidified to a pH below 2 using 100 μL of 37% HCl. The depolymerized mixture was filtered through a 0.22-micron PTFE filter prior to analysis by UPLC. Residence times were calculated by taking the ratio of the volume of the reactor and the flowrate:

$$Residence\ time\ (min) = \frac{V_{tube}(cm^3)}{Q_{pump\ flow\ rate}\ (cm^3 \cdot min^{-1})}$$

Table S2. Percent yields from aerobic alkaline oxidation of model compounds and RCF oligomers.

Substrate	**1**	**2**	**3**	**4**	**Pine RCF Oligomers***	**Poplar RCF Oligomers***
Peak Residence Time (s)	26	34	26	85	112	85
Vanillic acid	6.7	10.1	0	nd	2.8	1.7
Syringic acid	3.6	-	17.4	-	-	2.8
Vanillin	28.4	34.3	0	3.2	9.4	5.3
Syringaldehyde	18.3	-	25.8	-	-	10.9
Acetovanillone	7.1	5.6	0	nd	1.5	1.4
Acetosyringone	nd	-	16.8	-	-	2.9
p-Hydroxy benzaldehyde	-	-	-	-	0.5	-
p-Hydroxy benzoic acid	-	-	-	-	-	2.7
**5**	-	4.0	0	11.3	-	nd
**6**	-	4.4	0	4.9	1.8	1.4
**7**	-	7.4	0	6.0	3.2	5.5
Total Monomers	64	66	60	25	19	34

Compound **5** is 5-carboxyvanillic acid, **6** is 4-hydroxy-5-methoxyisophthaldehyde, and **7** is 5-formyl-2-hydroxy-3-methoxybenzoic acid. *wt% yield defined as g monomer/g RCF oligomer.

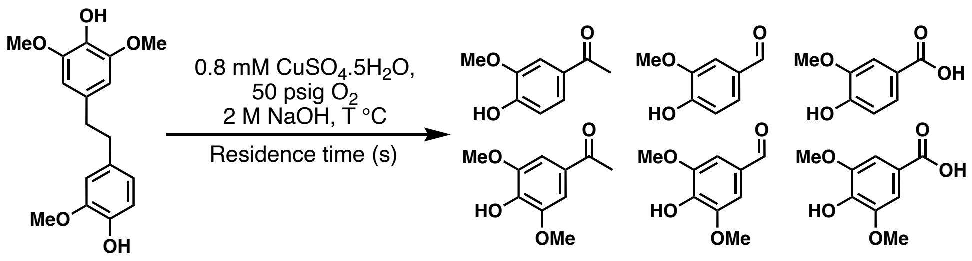

β-1 (1)

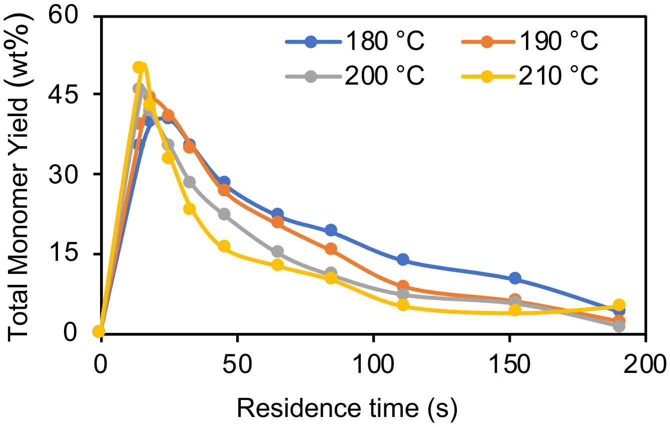

Figure S2. Temperature screen of cleavage of C-C bonds in oxidative alkaline depolymerization. Reaction conditions: 0.5 wt % β-□ substrate (**1**), 0.8 mM $CuSO_4$, 3.4 bar O_2, 2 M NaOH, and 1.5 mm ID PTFE membrane.

Oxidation of model compounds to assess stability procedure. The stock solutions were prepared using the following procedure: An approximately 5 mM solution of *p*HBA, vanillin, syringaldehyde, acetovanillone, vanillic acid, acetosyringone and syringic was prepared by dissolving the appropriate amount of compound in 2 M NaOH solution in a 100 mL volumetric flask. $CuSO_4 \cdot 5H_2O$ (0.020 g, 0.8 mM final concentration) was added followed by sonication before bringing to volume. For the individual component solutions, each analyte was prepared in a separate volumetric flask. In the mixture solution, all analytes were mixed and dissolved in a single volumetric flask. The Parr vessel was pressurized to 3.4 bar with oxygen gas with a gas outlet rate of ca. 1 bubble per second. For the flow reactor set-up, the back-pressure regulator was adjusted to 18 bar. Simultaneously, the flow reactor was preheated to the desired temperature (210 °C), and the HPLC pump was set to the required flow rate (0.24 mL/min to 4.50 mL/min), initially flowing deionized water to equilibrate the system. Once the reactor temperature stabilized, the pump was switched to draw from the stock solution. The stock solution was allowed to flow for at least three residence times before an aliquot was collected for sampling. The flowrate is controlled by an HPLC pump, and the volume of the reactor is calculated using the dimensions of the PTFE tube. A 0.5 mL aliquot collected after oxidation from the outlet was mixed with 0.5 mL of a 10 mM 1,4-dimethoxybenzene in methanol stock solution, used as an internal standard, and acidified to a pH below 2 using 100 µL of 37% HCl. The oxidized mixtures were filtered through a 0.22-micron PTFE filter into UPLC vials and analyzed using the UPLC. The concentrations were determined using a calibration curve.

Table S3. Oxidation of model compounds - individual components

Residence Time (sec)	*p*HBA (mM)	Vanillic Acid (mM)	Syringic acid (mM)	Vanillin (mM)	Syringaldehyde (mM)	Acetovanillone (mM)	Acetosyringone (mM)
0	5.3	4.5	4.7	4.9	5.4	5.2	5.1
10	5.2	3.9	2.6	4.8	4.9	4.7	3.3
13	5.0	3.4	1.9	4.6	4.7	4.5	2.5
15	5.0	3.1	1.5	4.4	4.5	4.3	1.9
17	4.9	2.5	1.0	4.4	3.8	4.1	1.8
26	4.6	1.5	0.0	4.2	2.9	2.2	0.2
34	4.6	0.9	0.0	4.1	2.0	0.4	0.0
47	4.4	0.6	0.0	3.6	1.1	0.2	0.0
66	3.3	0.5	0.0	3.0	0.3	0.1	0.0
85	3.0	0.5	0.0	3.0	0.1	0.0	0.0
112	2.8	0.6	0.0	3.0	0.0	0.0	0.0
153	2.9	0.4	0.0	2.7	0.0	0.0	0.0
192	2.8	0.4	0.0	3.0	0.1	0.0	0.0

Table S4. Oxidation of model compounds - mixed components

Residence Time (sec)	*p*HBA (mM)	Vanillic Acid (mM)	Syringic acid (mM)	Vanillin (mM)	Syringaldehyde (mM)	Acetovanillone (mM)	Acetosyringone (mM)
0	5.2	4.6	4.9	5.1	5.3	5.1	5.2
10	5.0	4.4	4.1	5.0	5.3	4.8	4.6
13	5.2	4.5	3.8	5.3	5.6	4.7	4.4
17	5.1	4.3	3.1	5.5	5.9	4.3	3.6
19	5.1	4.2	2.8	5.5	5.9	4.1	3.3
26	5.1	4.0	2.0	5.9	6.3	3.5	2.4
34	5.1	3.5	0.7	6.3	6.4	2.9	1.3
47	4.9	3.0	0.2	6.4	6.2	2.3	0.7
66	5.0	2.6	0.1	6.8	6.1	1.8	0.4
85	5.1	2.1	0.1	7.1	5.8	1.5	0.2
112	4.9	2.0	0.1	6.9	5.5	1.3	0.1
153	4.8	1.3	0.0	6.9	4.6	0.8	0.0
192	4.7	1.0	0.0	6.8	4.0	0.5	0.0

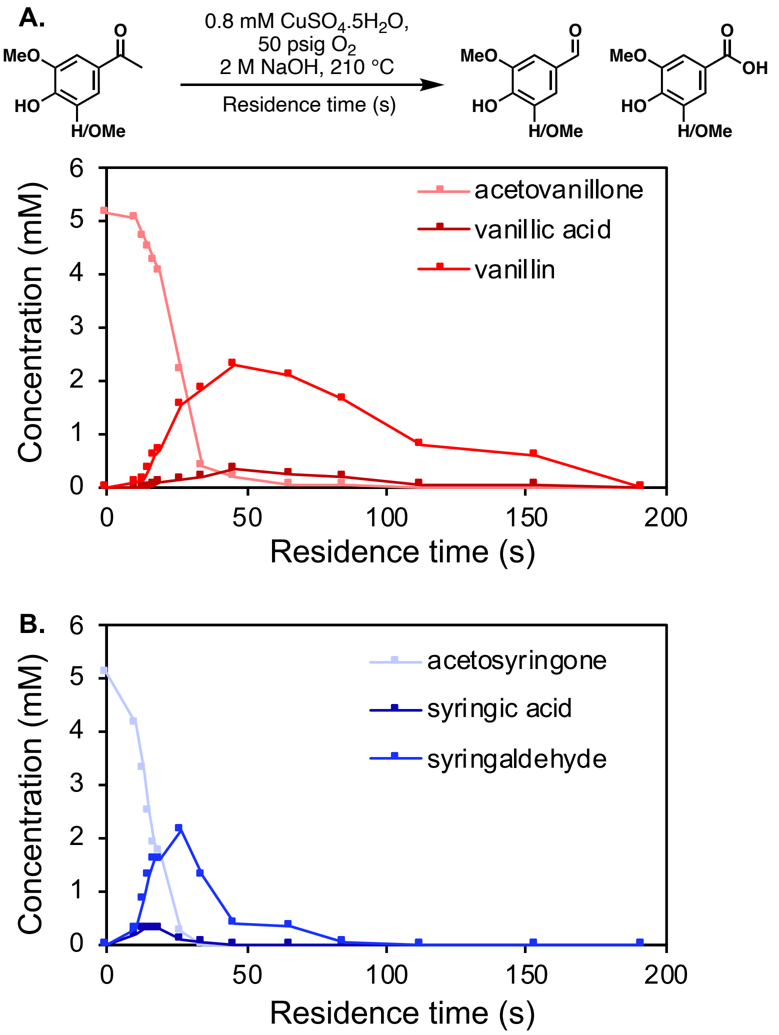

Figure S3. Acetovanillone is converted to vanillin and vanillic acid while acetosyringone is converted to syringaldehyde and syringic acid under alkaline and aerobic oxidation conditions.

Optimization of RCF Oligomer Oxidation in Flow

To optimize yields for real materials, pine and poplar derived RCF oligomers were subjected to oxidation in flow using the O_2-permeable membrane reactor at various residence times while varying temperature, O_2 pressure, Cu concentration (for pine oligomers), oligomer loading (for pine oligomers), and NaOH concentration (for poplar oligomers). The other conditions remained constant while a single variable were tested: 0.5 wt % oligomer, 0.8 mM $CuSO_4$, 3.4 bar O_2, 2 M NaOH. Cu concentration and NaOH concentration did not vary yields above 0.8 mM and 2 M, respectively, so were not varied in all cases to conserve material. Higher O_2 pressures improved oxidation rates while maintaining yield.

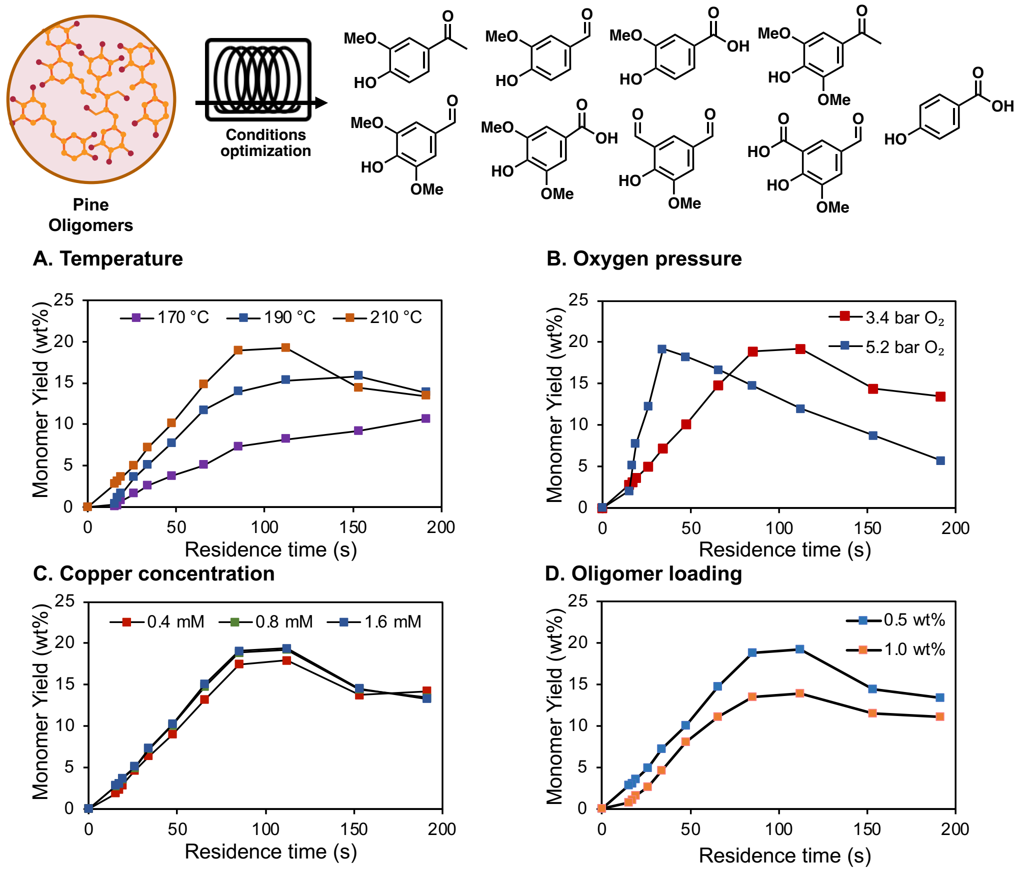

Figure S4. Optimization of flow reaction conditions using pine biomass derived RCF oligomers. Reaction conditions: 0.5 wt % oligomer, 0.8 mM $CuSO_4$, 3.4 bar O_2, 2 M NaOH unless the parameter of interest was varied. (A) Temperature impact on oligomer depolymerization. (B) Oxygen pressure impact on oligomer depolymerization. (C) Impact of copper catalyst concentration. (D) Oligomer loading impact on alkaline depolymerization.

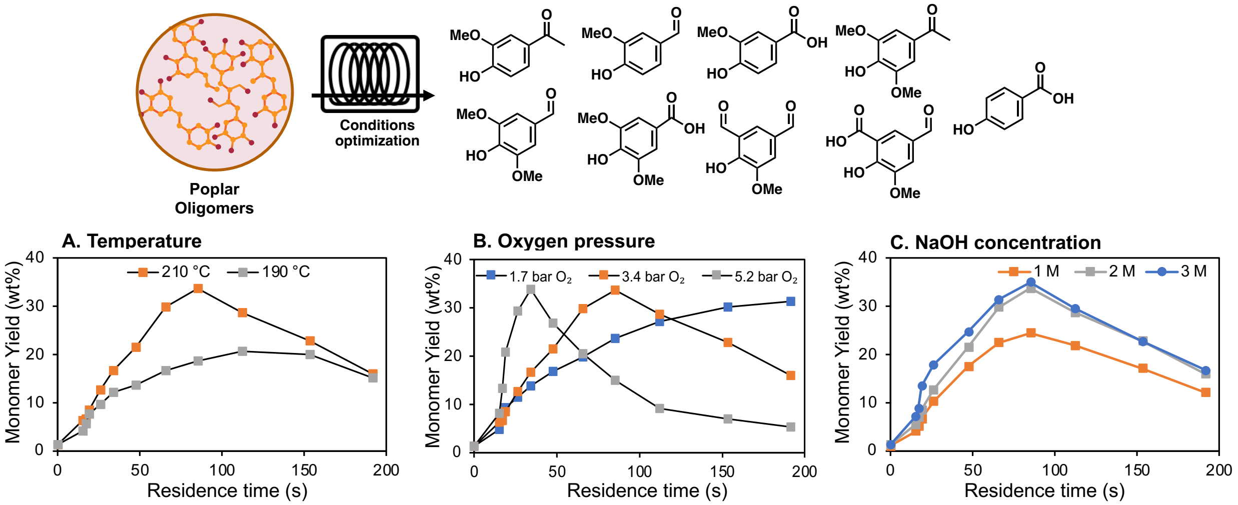

Figure S5. Optimization of flow reaction conditions using poplar biomass derived RCF oligomers. Reaction conditions: 210 ºC, 0.5 wt % oligomer, 0.8 mM $CuSO_4$, 3.4 bar O_2, 2 M NaOH unless the parameter of interest was varied. (A) Temperature impact on oligomer depolymerization. (B) Oxygen pressure impact on oligomer oxidation. (C) NaOH concentration effect on oligomer oxidation.

Bulk oxidation of oligomers

The pine oligomer solution contained 1.87 wt% of RCF monomers. The poplar oligomer solution contained 5.32 wt% of RCF monomers. Once an ideal residence time was found, the alkaline solution containing ~ 1 g each of the pine and poplar oligomer solutions at 0.5 wt% were flowed at a constant flow rate of 0.54 mL/min (85 s residence time). The alkaline solution containing the depolymerization products was collected and acidified to pH 2. The aqueous suspension was then extracted with a total of 100 mL ethyl acetate and evaporated to dryness giving 0.85 g oxidized pine oligomers (18 wt% monomer purity) and 1.00 g oxidized poplar oligomers (42.3 wt% monomer purity). The component assay is listed in **Table S5**.

Table S5. Assay of the product from bulk aerobic alkaline oligomer oxidation.

	Major Phenolic Products wt.%								
Retention time	Vanillic acid	Syringic acid	Vanillin	Syring-aldehyde	Aceto-vanillone	Aceto-syringone	*p*HBA[a]	**6**	**8**
**Pine oligomers**	2.6	-	11.2	-	1.6	-	0.3*	0.5	1.7
**Poplar oligomers**	2.6	4.2	8.3	17.5	1.2	3.3	4.1	-	1.0

[a]pHBA = *p*-hydroxybenzoic acid. Compound **6** is 4-hydroxy-5-methoxyisophthaldehyde, compound **7** is 5-formyl-2-hydroxy-3-methoxybenzoic acid. *denotes p-hydroxybenzaldehyde.

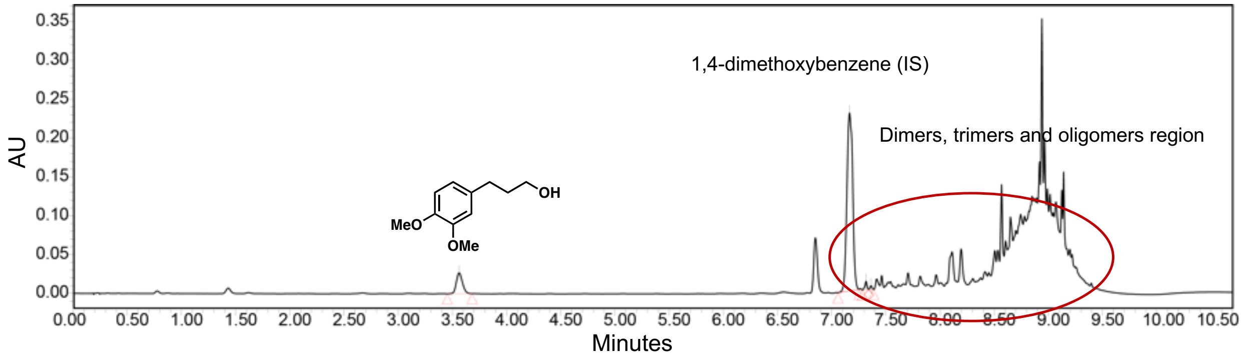

Figure S6. UPLC trace of pine oligomers before oxidation. 1,4-Dimethoxybenzene is used as an internal standard.

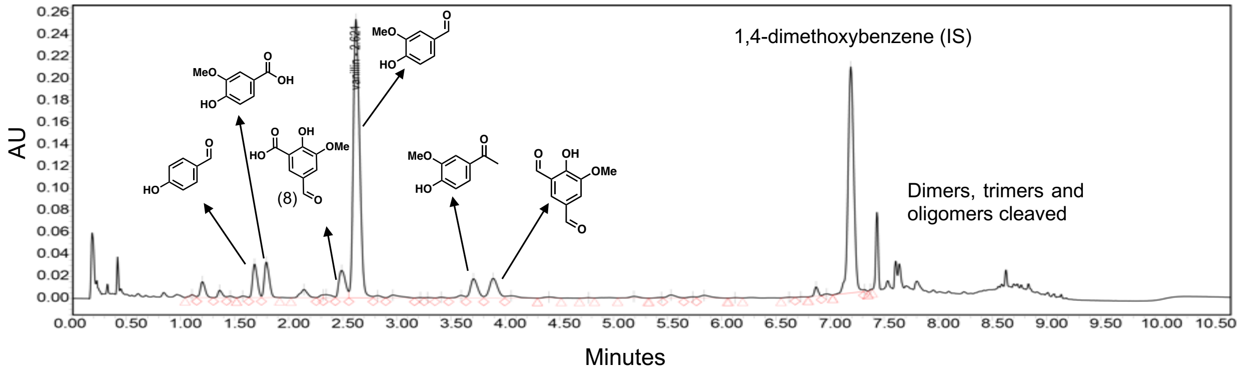

Figure S7. UPLC trace of pine oligomers after oxidation. 1,4-Dimethoxybenzene is used as an internal standard.

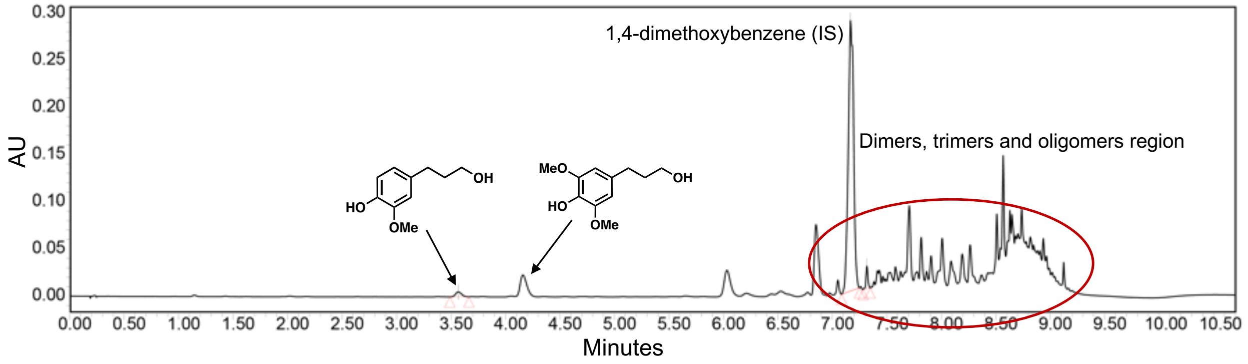

Figure S8. UPLC trace of poplar oligomers before oxidation. 1,4-Dimethoxybenzene is used as an internal standard.

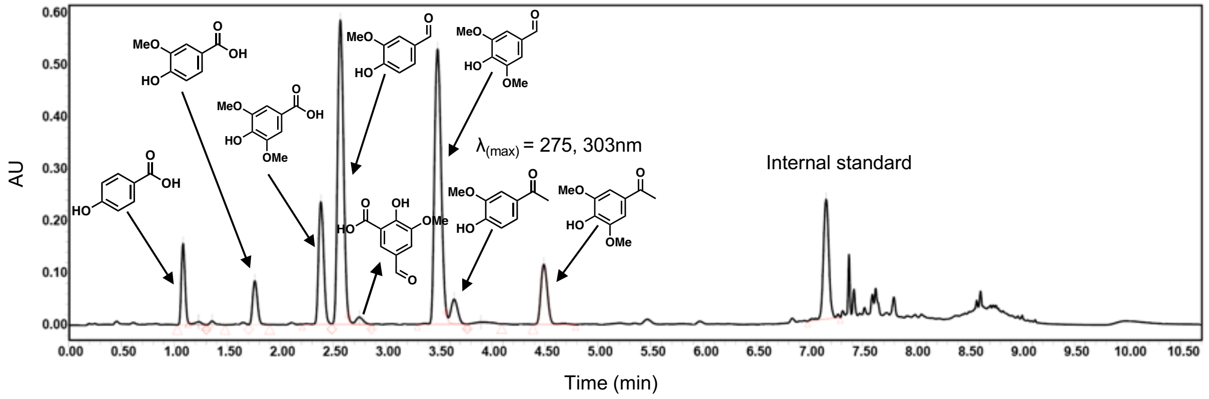

Figure S9. UPLC trace of poplar oligomers after oxidation. 1,4-Dimethoxybenzene is used as an internal standard.

Overall monomer yields

Table S6. Monomer yields from pine substrates (g monomer/100 g RCF oil before distillation)

Monomer	RCF oil	Oxidized RCF oligomers	Net monomer yield
4-Propanolguaiacol	7.2	-0.8[a]	6.4
4-Ethylguaiacol	0.7	0	0.7
Isoeugenol	2.2	0	2.2
4-Propylguaiacol	14.8	0	14.8
p-Hydroxybenzaldehyde	-	0.2	0.2
p-Hydroxybenzoic acid	-	0	0
Vanillic acid	-	1.2	1.2
Syringic acid	-	0	0
Vanillin	-	4.2	4.2
Syringaldehyde	-	0	0
Acetovanillone	-	0.7	0.7
Acetosyringone	-	0	0
**6**	-	0.8	0.8
**7**	-	1.4	1.4
**Total**	**24.9**	**7.7**	**32.6**

[a]Negative yield indicates monomers present in oligomer feedstock after distillation and therference were not recovered after oxidation.

Table S7. Monomer yields from poplar substrates (g monomer/100 g RCF oil before distillation)

Monomer	RCF oil	Oxidized RCF oligomers	Net monomer yield
4-Propanolguaiacol	9.4	-0.2[a]	9.2
4-Propanolsyringol	13.5	-2.0[a]	11.5
Phenol	3.2	0	3.2
4-Ethylguaiacol	0.3	0	0.3
Isoeugenol	0	0	0
4-Propylguaiacol	6.5	0	6.5
4-Propylsyringol	14.0	-0.1[a]	13.9
Methyl paraben	1.4	0	1.4
p-Hydroxybenzaldehyde	-	0.1	0.1
p-Hydroxybenzoic acid	-	1.2	1.2
Vanillic acid	-	0.8	0.8
Syringic acid	-	1.2	1.2
Vanillin	-	2.3	2.3
Syringaldehyde	-	4.8	4.8
Acetovanillone	-	0.6	0.6
Acetosyringone	-	0.8	0.8
**6**	-	0.6	0.6
**7**	-	2.4	2.4
**Total**	**48.3**	**12.5**	**60.7**

[a]Negative yield indicates monomers present in oligomer feedstock after distillation that were not recovered after oxidation.

6. Biological Conversion

Preparation of lignin oxidation oils for bioconversion: Aromatics mixture from poplar and pine wood were solubilized in pure ethanol at 250 mM total lignin-derived monomers. Mock solutions of the deconstructed lignin oils were prepared by dissolving commercially purchased aromatic monomers (vanillate, vanillin, 4-hydroxybenzoate, syringate, and syringaldehyde) in ethanol at 250 mM (total

monomers) in the same ratios as those found in the poplar- and pine-derived oils. Solubilized deconstructed lignin oils and mock solutions were added to M9 minimal medium (6.78 g/L Na_2HPO_4, 3 g/L KH_2PO_4, 0.5 g/L NaCl, 1 g/L NH_4Cl, 2 mM $MgSO_4$, 100 µM $CaCl_2$, and 18 µM $FeSO_4$, pH 7.0) containing 40 mM glucose (AW045 cultures) or 10 mM glucose (CJ781 cultures) at final concentrations of 5 mM (2% ethanol) aromatic monomers.

Bacterial strains, media, and cultivation conditions: *Pseudomonas putida* KT2440 derived strains (**Table S6**) were revived from glycerol stocks (20% v/v) by inoculating into 25 mL of LB broth (Miller) and cultivating overnight (~12-16 h) at 30 ºC and 225 rpm. Cells were washed with 1 x M9 salts and inoculated in triplicate into 25 mL of M9 minimal medium (described above) containing 5 mM of aromatic monomers as indicated in each experiment at an optical density at 600 nm (OD_{600}) of 0.1. Cells were cultivated in 125 mL baffled Erlenmeyer flasks with metal caps at 30 °C and 225 rpm in a benchtop incubator (0.75" orbital). AW045 cultures were fed 20 mM glucose every 24 hours while CJ781 cultures were fed 10 mM glucose every 12 hours. Growth was measured via OD_{600} using a 1:10 dilution. To sample for metabolite analysis, 0.8 mL of culture was removed, centrifuged for 2 min at >18,000*g,* and filtered (0.2 µm syringe filter) into amber glass vials. Samples were stored at -20 °C prior to analysis.

Analysis of bioconversion metabolites. Quantification of PDC, muconate and aromatic acids were analyzed by UHPLC-DAD as previously described. Glucose and small aliphatic coproducts were analyzed by HPLC as previously detailed.[8,9] Briefly, samples and standards were injected onto and Aminex HPX-87H column (Bio-Rad) and eluted using an isocratic mobile phase of 0.01 N sulfuric acid in water. A refractive index detector was utilized for detection and quantitation. Molar product yields were calculated by dividing the moles of product at 36 h (corrected for sampling volumes changes) by the moles of total bioavailable monomers at 0 h and multiplying by 100%. Bioavailable monomers were considered to be those with native or engineered pathways (VA, Vn, SA, SAL, and 4HBA).

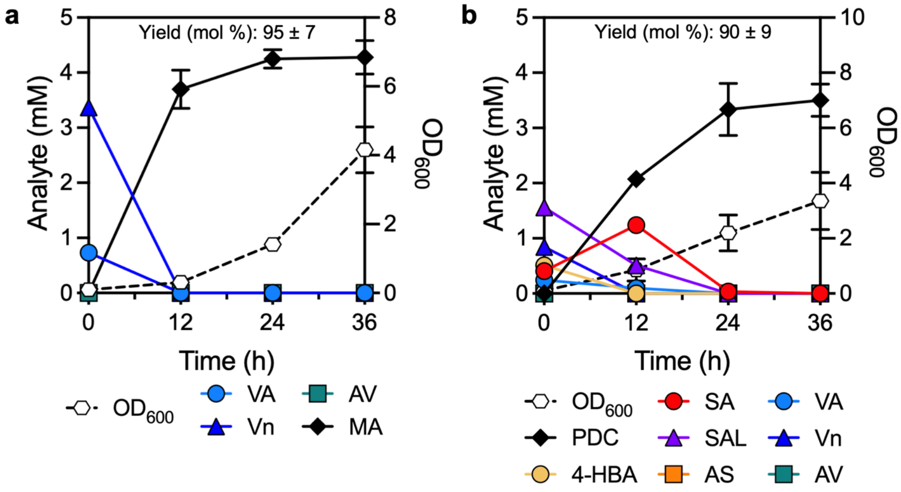

Figure S10. Bioconversion of model aromatic monomers to (**a**) muconate or (**b**) PDC. (**a**) CJ781 and (**b**) AW045 were cultivated in shake flasks at 30 ºC/225 rpm in M9 minimal medium supplemented with a) 10 mM glucose and a mock solution of pine-derived deconstructed lignin-oil or b) 40 mM glucose and a mock solution of poplar-derived deconstructed lignin oil. CJ781 cultivations were fed to 10 mM glucose at 12, 24, and 48 h. AW045 cultivations were fed to 20 mM glucose every 24 h. Chemical abbreviations: VA, vanillate; Vn, vanillin; AV, acetovanillone; MA, muconate; 4-HBA, 4-hydroxybenzoate; SA, syringate; SAL, syringaldehyde; AS, acetosyringone; 2-pyrone-4,6-dicarboxylic acid, PDC; OD_{600}, optical density, measured as absorbance at 600 nm; h: hours. Data points and error bars represent the mean and standard deviation, respectively, of n = 3 biological replicates.

Table S8. Bacterial strains utilized in this study.

Strain name	Genotype	Reference
AW045	*Pseudomonas putida* KT2440 Δ*pcaHG*::P_{tac}:*ligABC*$_{\mathrm{SYK6}}$ Δ*vanAB*::P_{tac}:*vanAB*$_{\mathrm{HR199}}$	10
CJ781	*Pseudomonas putida* KT2440 Δ*catRBCA*::P_{tac}:*catA* Δ*pcaHG*::P_{tac}:*aroY:ecdBD* Δ*crc* Δ*pobAR fpvA*::P_{tac}:*praI:vanAB*	11

7. Analytical Methods

Gel permeation chromatography analysis: 15-20 mg of hydrogenolysis lignin oligomers or oxidized oligomer samples were diluted in THF and stirred for 30 minutes. The THF solution was filtered through a 0.2 µm syringe filter into an HPLC vial. For analysis, 20 µL of the sample was injected into an HPLC system equipped with three PLgel 7.5 x 300 mm columns in series: 10 µm x 50 Å, 10 µm x 103 Å, 10 µm x 104 Å (Agilent Technologies, Stockport, UK). The analysis was performed at ambient temperature using an isocratic flow of 100% tetrahydrofuran (Sigma-Aldrich inhibitor-free ≥ 99.9%) at a rate of 1 mL/min for 45 minutes. Analytes were monitored at wavelengths of 210 nm, 260 nm, and 270 nm using a Diode Array Detector (DAD).

Molecular weight calibrations were performed using five polystyrene standards:

PS1 = 677,500 Da, 68,000 Da, 5,050 Da, 950 Da, 92 Da
PS2 = 465,000 Da, 34,500 Da, 3,600 Da, 580 Da, 92 Da
PS3 = 1,820,000 Da, 369,500 Da, 22,000 Da, 3,250 Da, 92 Da
PS4 = 2,200,000 Da, 271,800 Da, 11,600 Da, 1,700 Da, 92 Da
PS5 = 980,000 Da, 170,000 Da, 9,200 Da, 1,250 Da, 92 Da

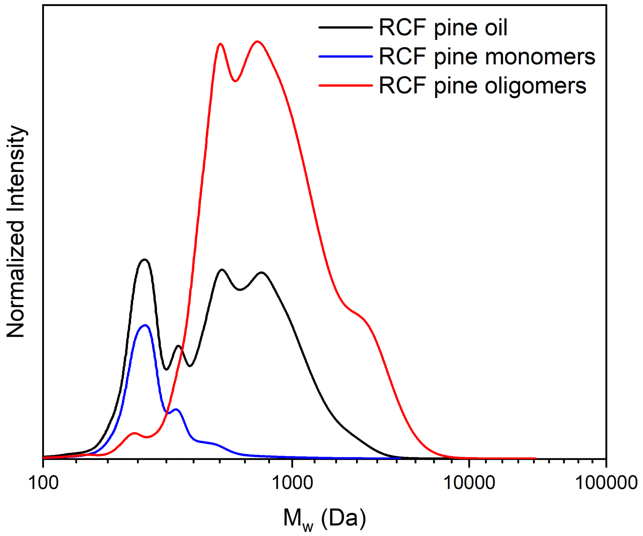

Figure S11. GPC Traces of RCF pine oil, pine oligomers, and RCF pine monomers.

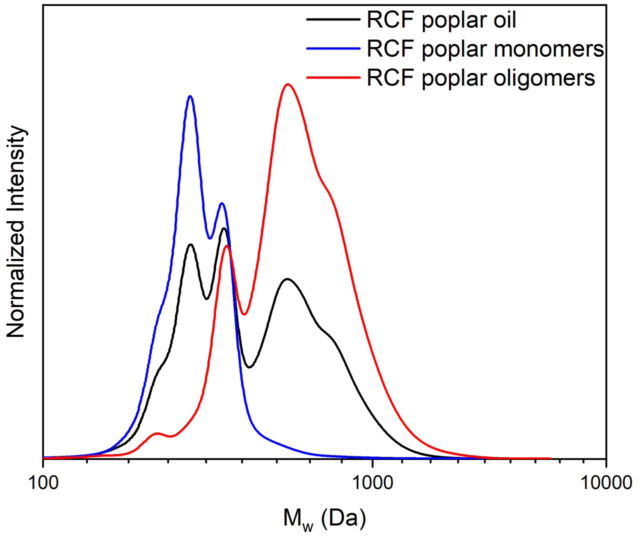

Figure S12. GPC Traces of RCF poplar oil, poplar oligomers, and RCF poplar monomers.

UPLC quantification of aromatic monomers and dimers: UPLC analysis was done using Waters Acquity class H QSM plus UPLC equipped with a BEH C18 1.7 µm, 2.1 x 50 mm column using a photodiode array (PDA) detector. Data were acquired using Empower software. Calibration curves of model compounds **1-4** and products were obtained by injection of solutions after serial dilution of a stock solution of the analytes. The compound to be analyzed was dissolved in a minimal quantity of methanol, transferred to a 10 mL volumetric flask. The contents of the volumetric flask were diluted to the mark and agitated to ensure solution homogeneity. The solution was filtered through a 0.45-micron PTFE syringe filter and 500 µL were transferred to a UPLC vial. A separate 10 mM internal standard solution was made by dissolving 690.8 mg (5 mmol) of 1,4-dimethoxybenzene in methanol and diluting to the mark of a 500 mL volumetric flask. Every sample added to a UPLC vial to be analyzed was further diluted with internal standard solution such that the final concentration of the analytes and internal standard is between the lowest and highest standards in the calibration curves, where the detector response is linear. The internal standard was added to the samples to correct the monomer concentrations by accounting for potential UPLC system variability. Using the specified gradient profile (Table S9), calibration curves were generated with peak integration at 280 nm (±0 bandwidth).

For the monomer products, a calibration curve stock solution was generated by adding the materials to a 25 mL volumetric flask, dissolving the mixed sample, and filling the volumetric flask to the mark with methanol to achieve a final concentration of each solute between 15 – 22 mM. Serial dilutions of the stock solution were done to achieve various intermediate concentrations for the analytes, each filtered through a 0.45-micron syringe filter. Then, 500 µL of each filtered solution was added to a UPLC vial containing 500 µL of a 10 mM 1,4-dimethoxy benzene internal standard solution.

Samples were eluted using 0.1% formic acid in Milli-Q water and UPLC grade methanol as the A and B mobile phases respectively. Beginning at 3% B, the mobile phase was pumped at 0.75 mL/min with the following gradient profile:

Table S9. UPLC gradient profile for aromatics quantitation.

Time (mins)	Flow (mL/min)	%A	%B
Initial	0.750	97.0	3.0
0.30	0.750	97.0	3.0
6.00	0.750	87.5	12.5
7.00	0.750	70.0	30.0
7.80	0.750	65.0	35.0
8.70	0.750	10.0	90.0
9.00	0.750	10.0	90.0
10.00	0.750	97.0	3.0
10.70	0.750	97.0	3.0

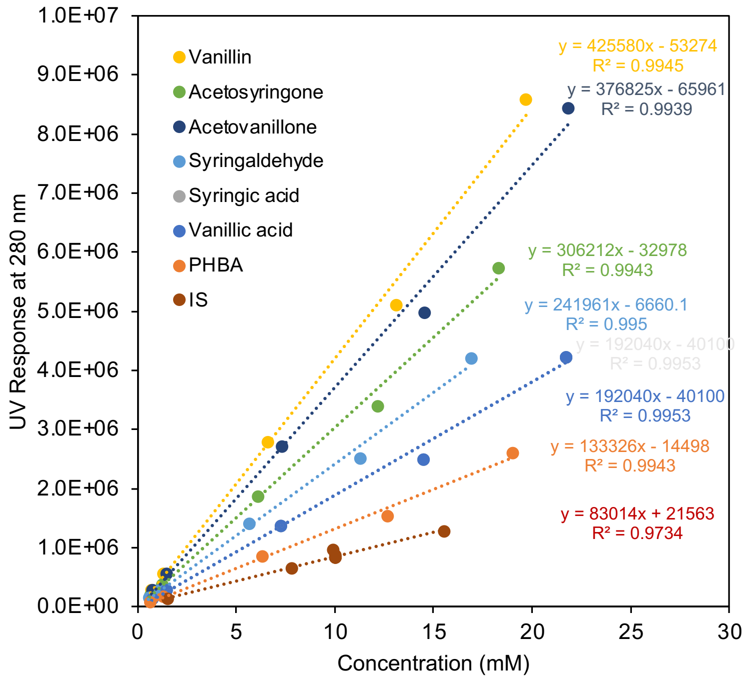

Figure S13. Calibration curves and linear fit equations were made for all the major aromatics obtained from oligomers oxidation.

Aromatics yields in weight percent (wt.%) were computed as the fraction of mass of monomers by UPLC to the mass of lignin:

$$\text{Aromatics Yield (wt.\%)} = \frac{\text{mass of aromatics by UPLC (mg)}}{\text{mass of isolated lignin added (mg)}} \times 100$$

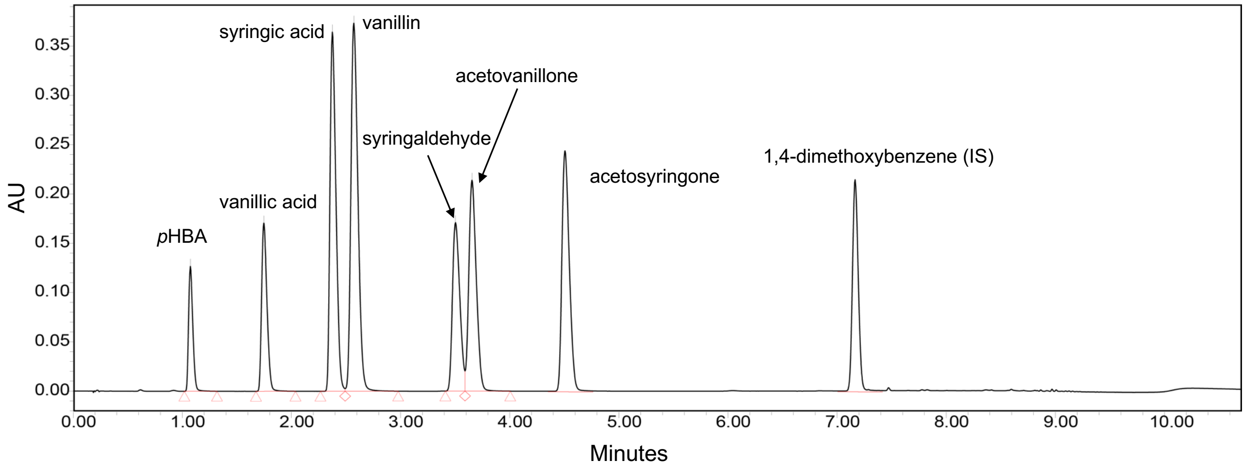

Figure S14. UPLC trace with retention times of model aromatics typically obtained from the cleavage of oligomers under alkaline and aerobic conditions. The internal standard used for the quantification is also shown.

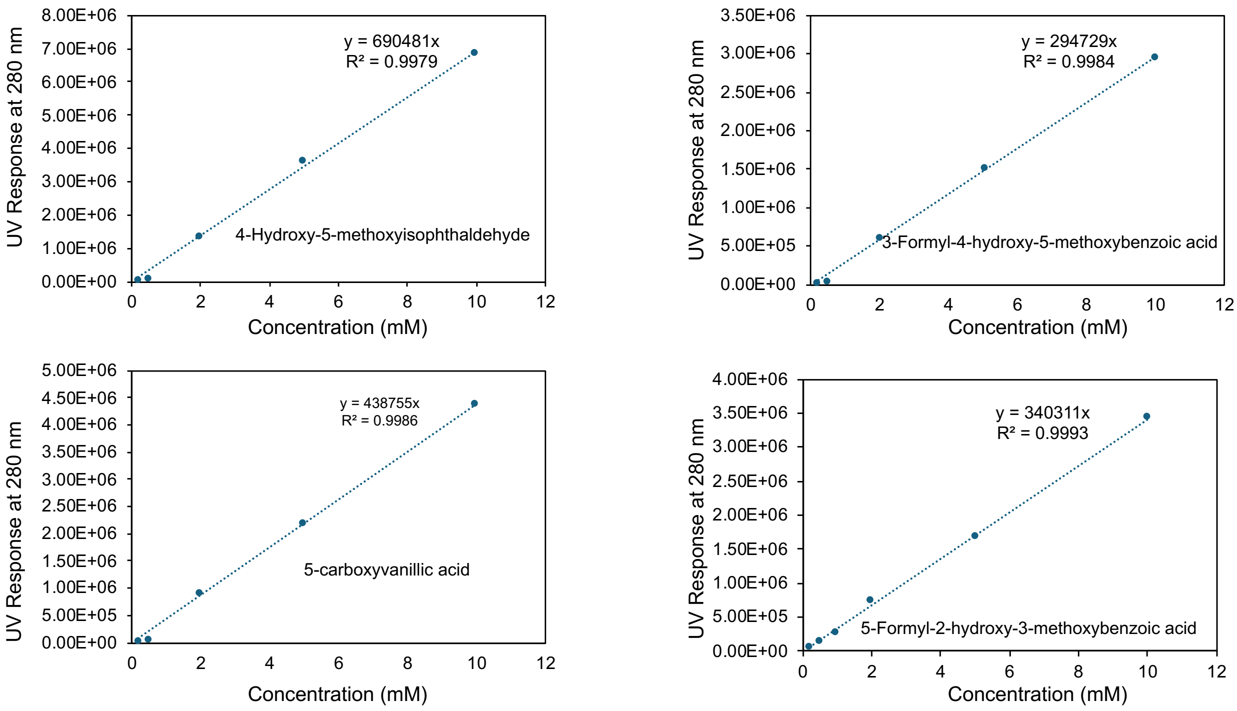

Figure S15. Calibration curves and linear fit equations for 4-hydroxy-5-methoxyisophthaldehyde, 3-formyl-4-hydroxy-5-methoxybenzoic acid, 5-carboxyvanillic acid, 5-formyl-2-hydroxy-3-methoxybenzoic acid.

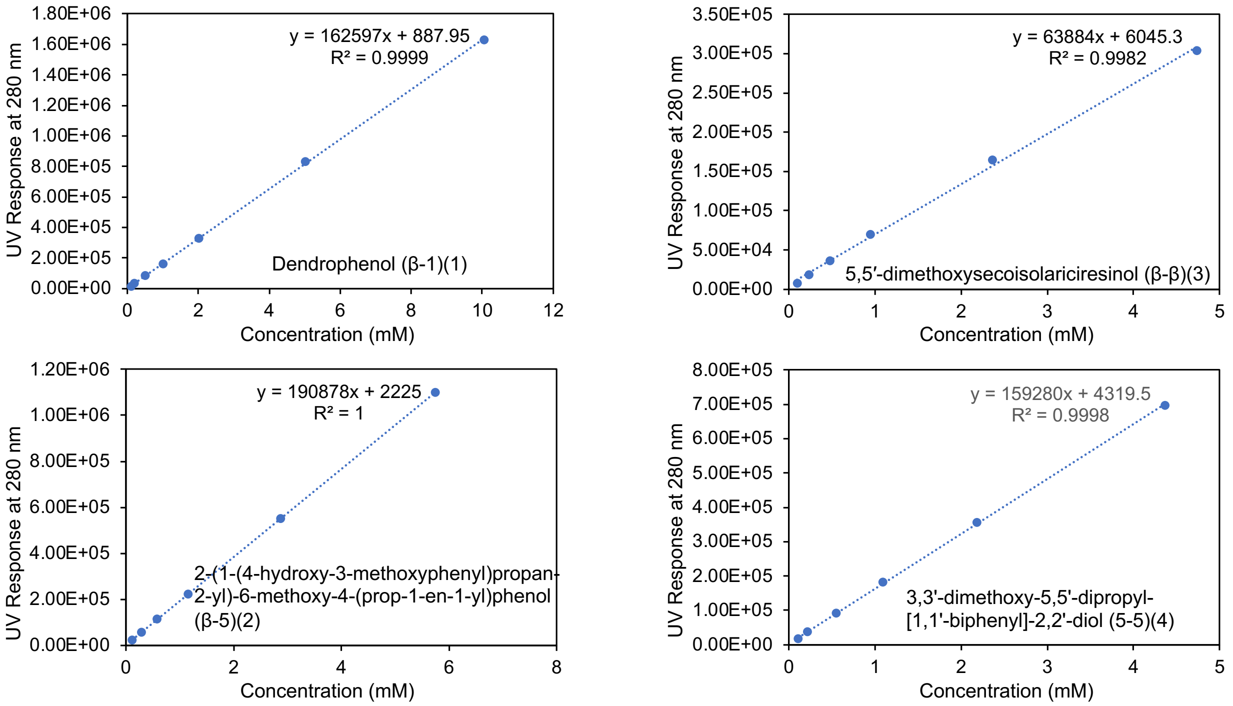

Figure S16. Calibration curves and linear fit equations for all the dimeric model compounds. Top left (β-1 model **1**), top right (β-5 model **2**), bottom left (β-β model **3**) and bottom right (5-5 model **4**).

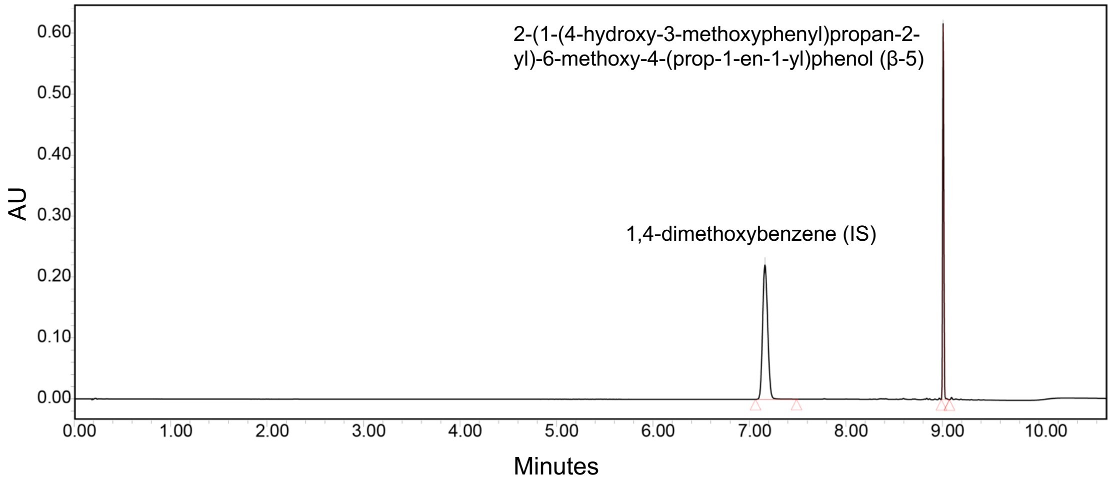

Figure S17. UPLC trace, with retention time of β-5 dimer **1**. 1,4-Dimethoxybenzene is used to correct the concentration of the β-5 dimer.

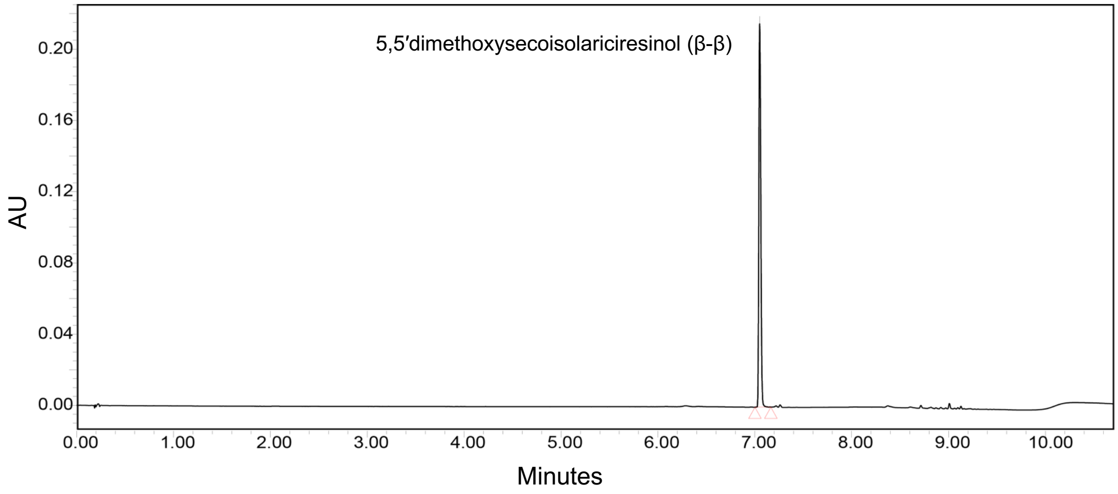

Figure S18. UPLC trace, with retention time of β-β dimer **2**. The internal standard and associated correction was not used in the quantitation of the β-β-dimer because both peaks co-elute.

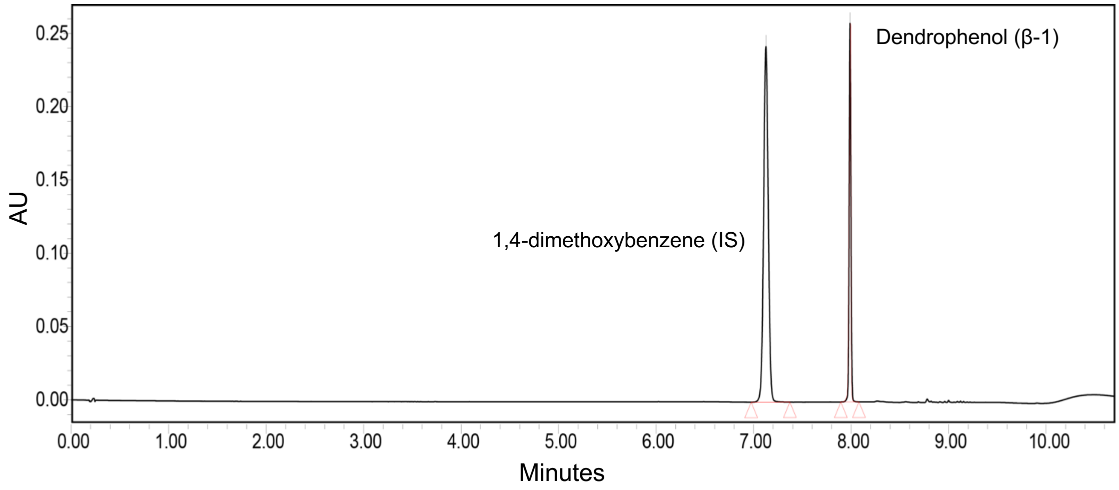

Figure S19. UPLC trace, with retention time of β-1 dimer **3**. 1,4-Dimethoxybenzene is used to correct the concentration of the β-1 dimer.

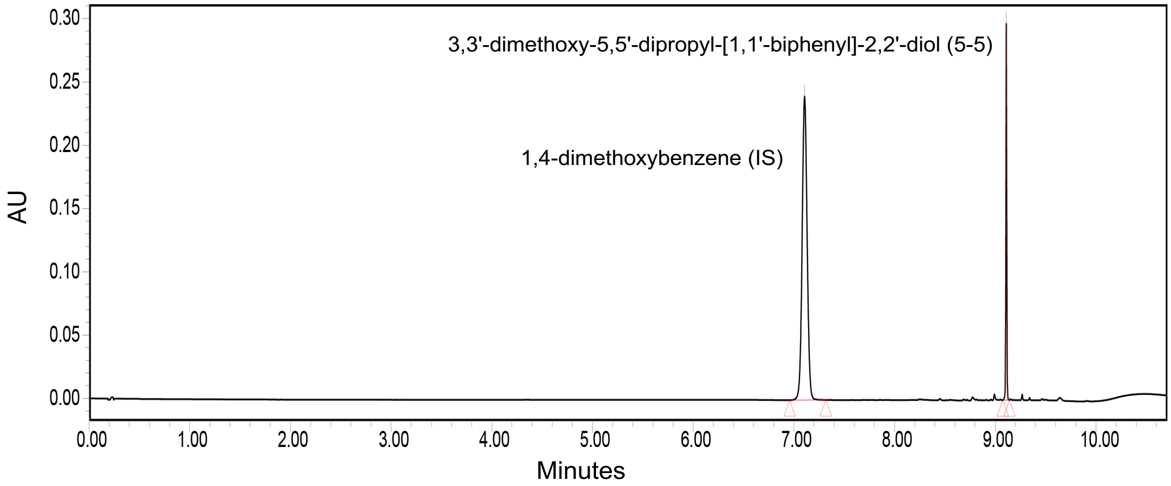

Figure S20. UPLC trace, with retention time of 5-5 dimer **4**. 1,4-Dimethoxybenzene is used to correct the concentration of the 5-5 dimer.

8. NMR and Mass Spectra

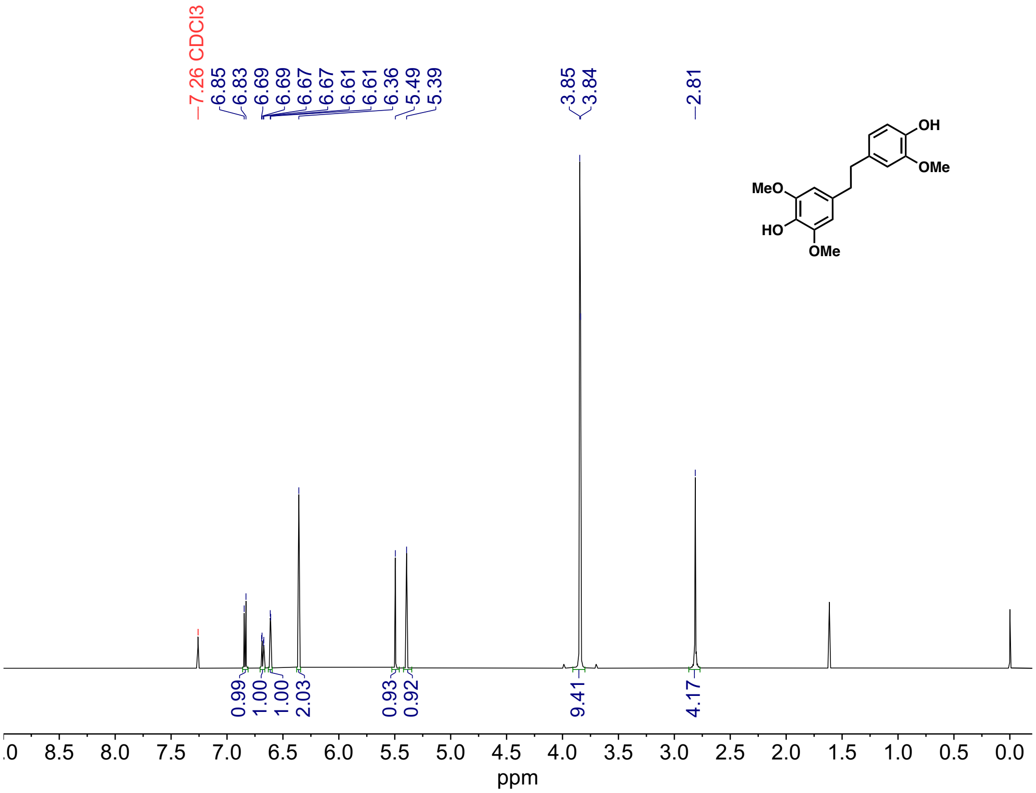

Figure S21. ^{1}H NMR spectrum of dendrophenol (**1**) in $CDCl_3$.

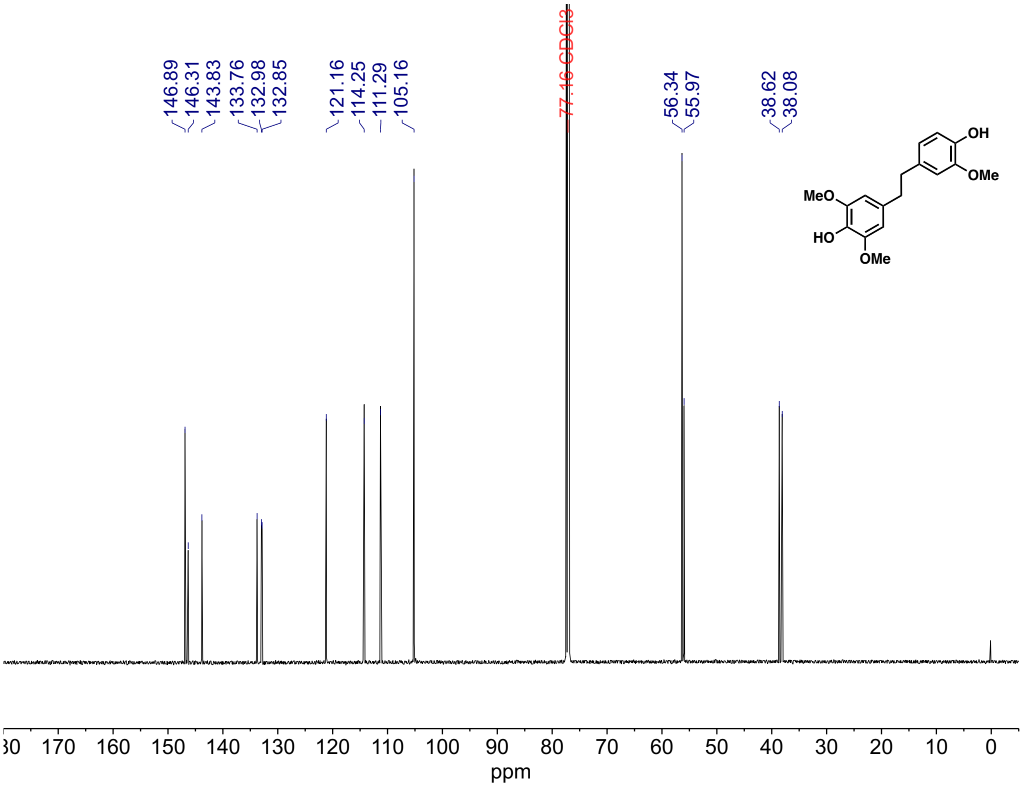

Figure S22. ^{13}C NMR spectrum of dendrophenol (**1**) in $CDCl_3$.

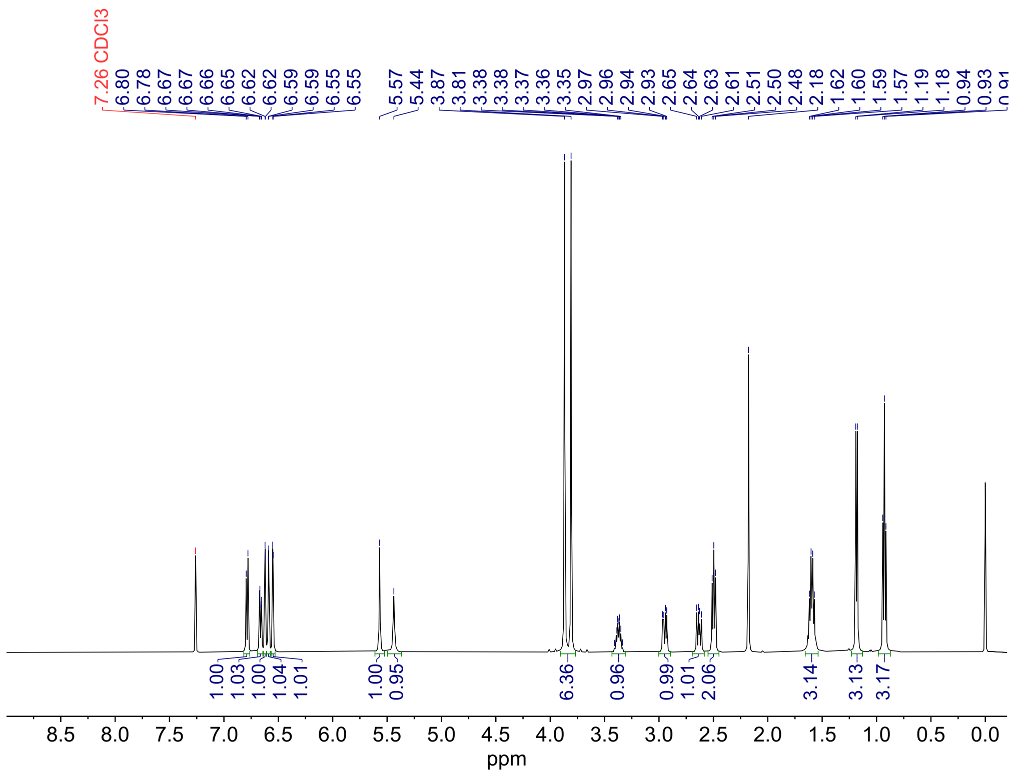

Figure S23. ^{1}H NMR spectrum of 2-(1-(4-hydroxy-3-methoxyphenyl)propan-2-yl)-6-methoxy-4-(prop-1-en-1-yl)phenol (**2**) in $CDCl_3$.

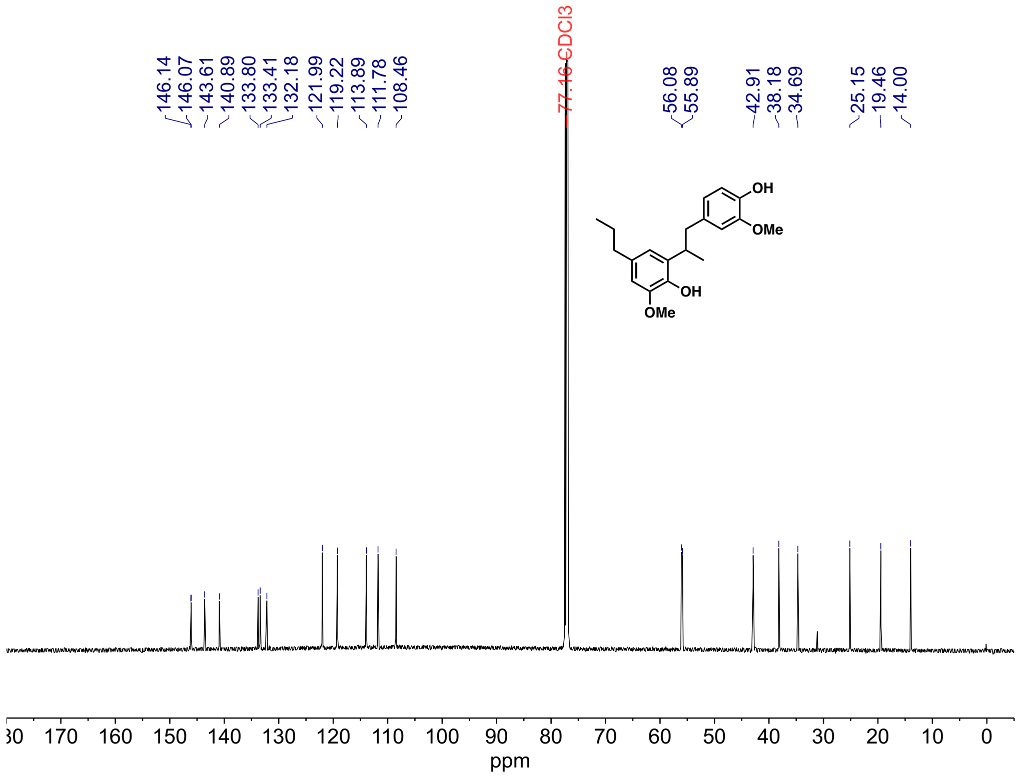

Figure S24. ^{13}C NMR spectrum of 2-(1-(4-hydroxy-3-methoxyphenyl)propan-2-yl)-6-methoxy-4-(prop-1-en-1-yl)phenol (**2**) in $CDCl_3$.

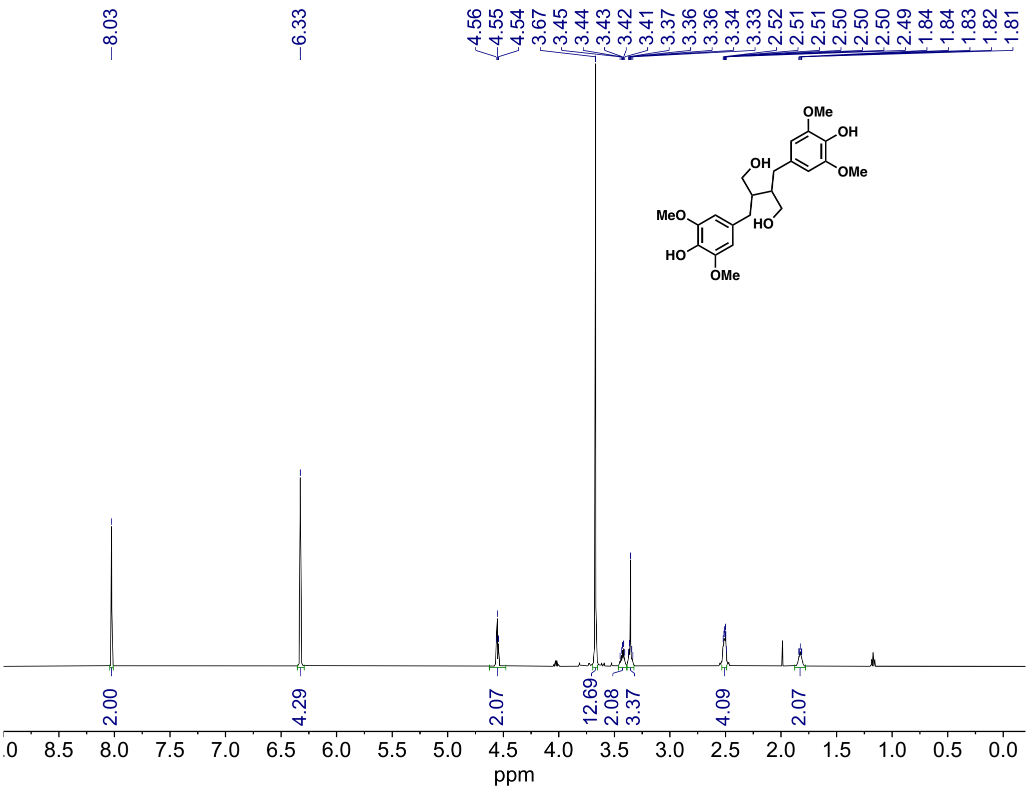

Figure S25. ^{1}H NMR spectrum of 5,5′-dimethoxysecoisolariciresinol (**3**) in DMSO-d_6.

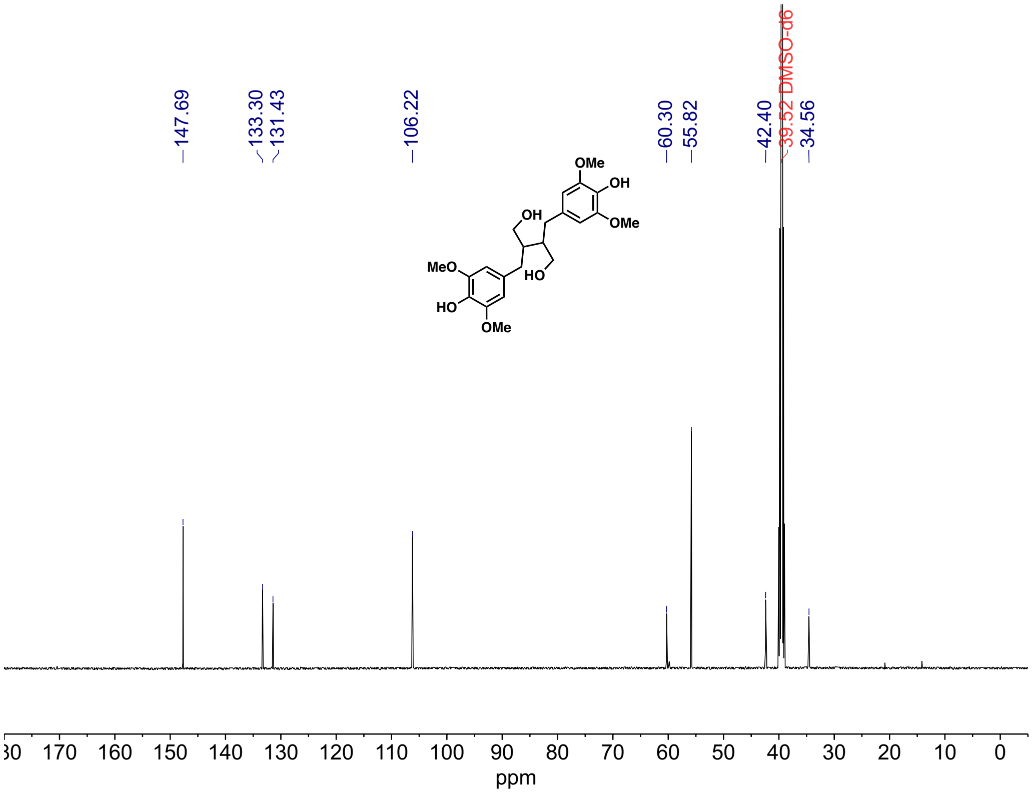

Figure S26. ^{13}C NMR spectrum of 5,5′-dimethoxysecoisolariciresinol (**3**) in DMSO-d_6.

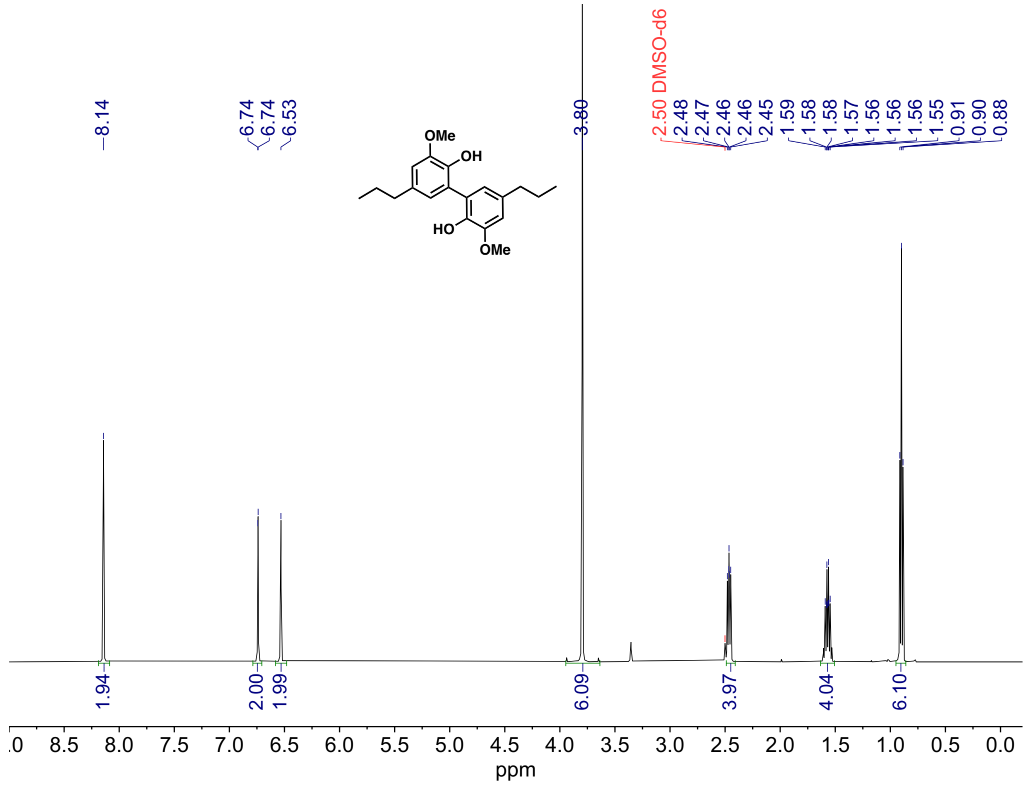

Figure S27. ^{1}H NMR spectrum of 3,3'-dimethoxy-5,5'-dipropyl-[1,1'-biphenyl]-2,2'-diol (**4**) in DMSO-d_6.

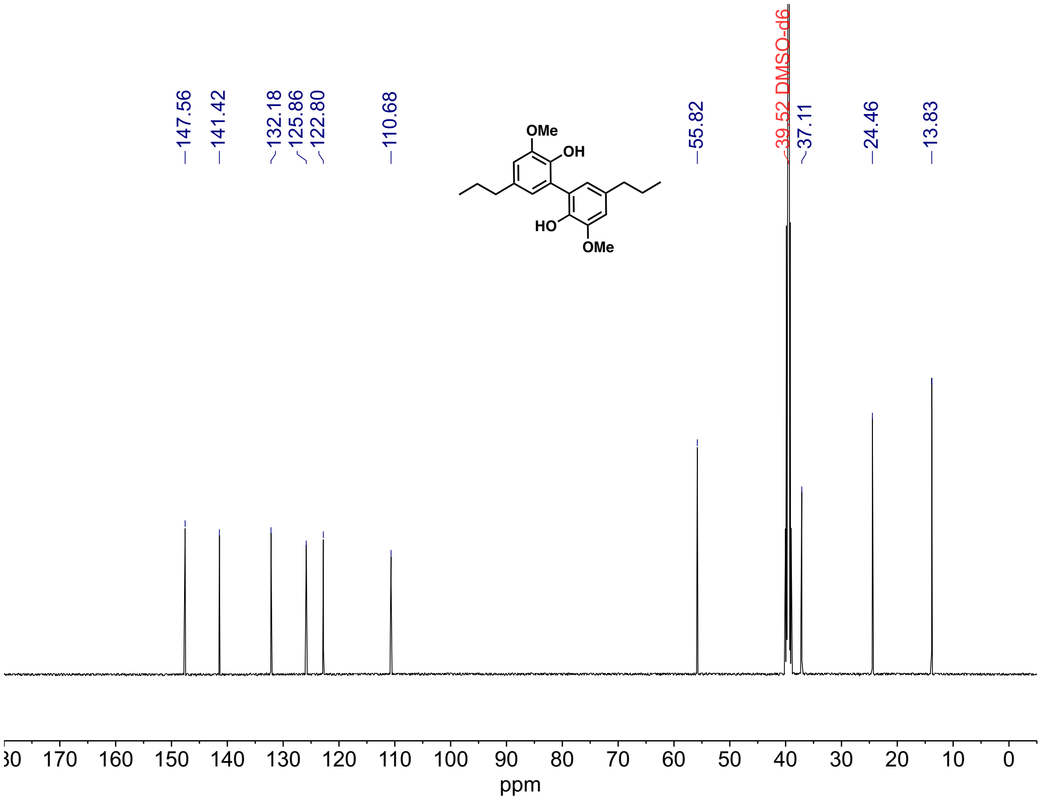

Figure S28. ^{13}C NMR spectrum of 3,3'-dimethoxy-5,5'-dipropyl-[1,1'-biphenyl]-2,2'-diol (**4**) in DMSO-d_6.

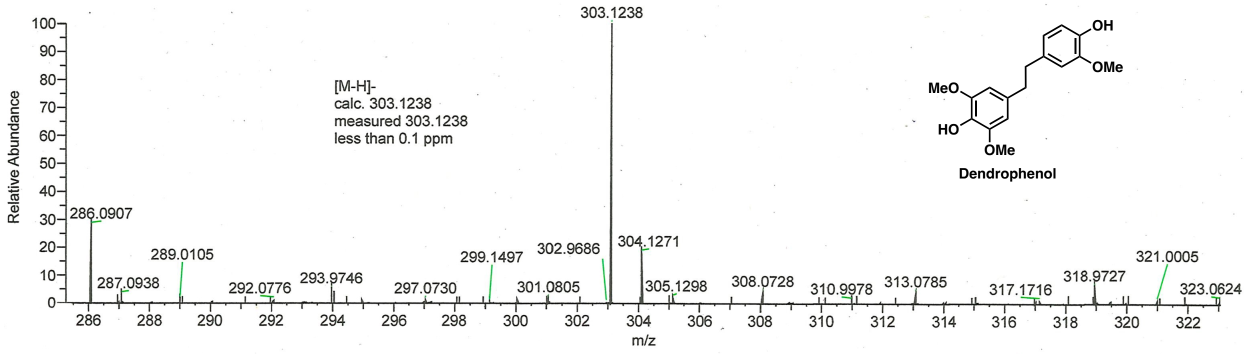

Figure S29. HRMS of dendrophenol (**1**).

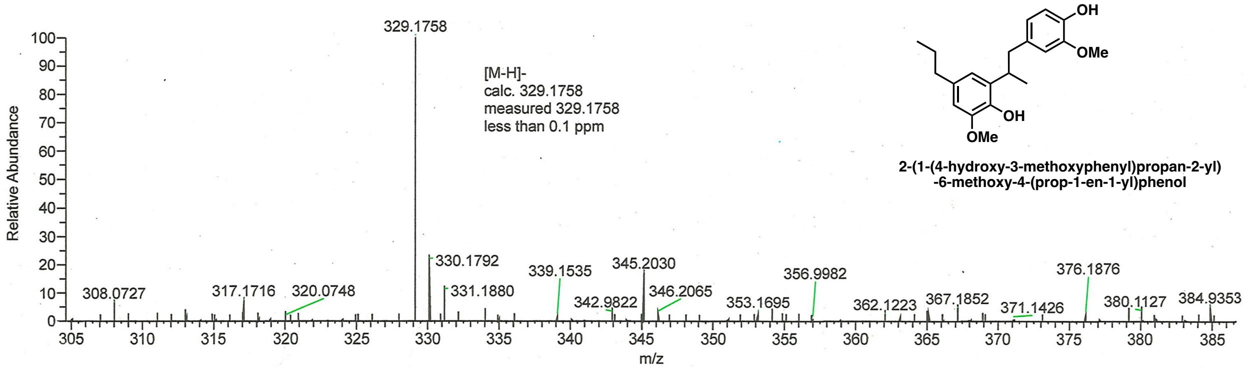

Figure S30. HRMS of 2-(1-(4-hydroxy-3-methoxyphenyl)propan-2-yl)-6-methoxy-4-(prop-1-en-1-yl)phenol (β-5 dimer) (**2**).

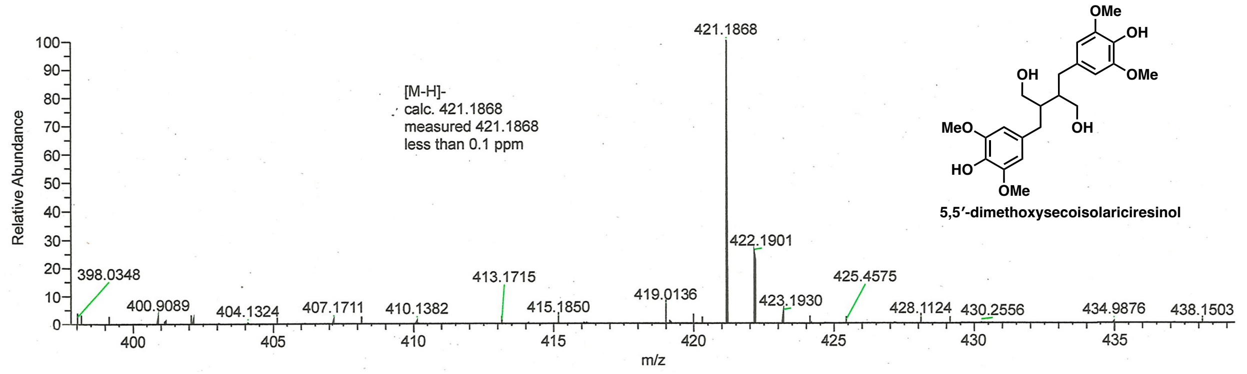

Figure S31. HRMS of 5,5′-dimethoxysecoisolariciresinol (**3**).

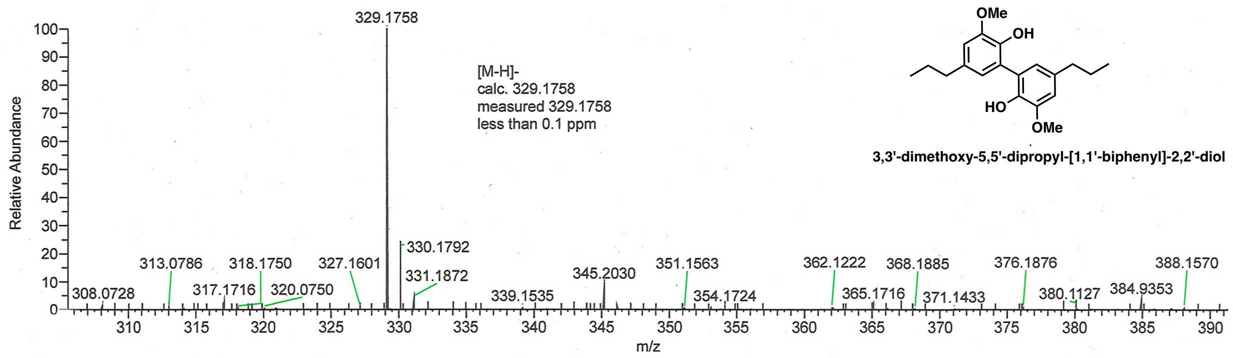

Figure S32. HRMS of 3,3'-dimethoxy-5,5'-dipropyl-[1,1'-biphenyl]-2,2'-diol (**4**).

9. References

(1) Weeda, E. P.; Holland, C. M.; Bueren, J. B. de; Yuan, Z.; Alherech, M.; Coplien, J.; Haak, D.; Hegg, E. L.; Luterbacher, J.; Root, T. W.; Stahl, S. S. O_2-Permeable Membrane Reactor for Continuous Oxidative Depolymerization of Lignin. *Joule* **2024**, *8* (12), 3336–3346. https://doi.org/10.1016/j.joule.2024.08.015.

(2) Jang, H. Y.; Park, H. J.; Damodar, K.; Kim, J.-K.; Jun, J.-G. Dihydrostilbenes and Diarylpropanes: Synthesis and in Vitro Pharmacological Evaluation as Potent Nitric Oxide Production Inhibition Agents. *Bioorg. Med. Chem. Lett.* **2016**, *26* (22), 5438–5443. https://doi.org/10.1016/j.bmcl.2016.10.034.

(3) Lancefield, C. S.; Westwood, N. J. The Synthesis and Analysis of Advanced Lignin Model Polymers. *Green Chem.* **2015**, *17* (11), 4980–4990. https://doi.org/10.1039/C5GC01334H.

(4) Yue, F.; Lu, F.; Regner, M.; Sun, R.; Ralph, J. Lignin-Derived Thioacidolysis Dimers: Reevaluation, New Products, Authentication, and Quantification. *ChemSusChem* **2017**, *10* (5), 830–835. https://doi.org/10.1002/cssc.201700101.

(5) Quideau, Stephane.; Ralph, John. Facile Large-Scale Synthesis of Coniferyl, Sinapyl, and p-Coumaryl Alcohol. *J. Agric. Food Chem.* **1992**, *40* (7), 1108–1110. https://doi.org/10.1021/jf00019a003.

(6) Rahman, MD. A.; Katayama, T.; Suzuki, T.; Nakagawa, T. Stereochemistry and Biosynthesis of (+)-Lyoniresinol, a Syringyl Tetrahydronaphthalene Lignan in Lyonia Ovalifolia Var. Elliptica I: Isolation and Stereochemistry of Syringyl Lignans and Predicted Precursors to (+)-Lyoniresinol from Wood. *J. Wood Sci.* **2007**, *53* (2), 161–167. https://doi.org/10.1007/s10086-006-0832-1.

(7) Uyanik, M.; Nagata, D.; Ishihara, K. Hypoiodite-Catalysed Oxidative Homocoupling of Arenols and Tandem Oxidation/Cross-Coupling of Hydroquinones with Arenes. *Chem. Commun.* **2021**, *57* (88), 11625–11628. https://doi.org/10.1039/D1CC05171G.

(8) Alt, H. M.; Benson, A. F.; Haugen, S. J.; Ingraham, M. A.; Michener, W. E.; Woodworth, S. P.; Ramirez, K. J.; Beckham, G. T. Analysis of Sugars, Small Organic Acids, and Alcohols by HPLC-RID. *protocols.io* **2024**. https://doi.org/dx.doi.org/10.17504/protocols.io.5qpvob7y9l4o/v2.

(9) Woodworth, S. P.; Haugen, S. J.; Michener, W. E.; Ramirez, K. J.; Beckham, G. T. Muconic Acid Isomers and Aromatic Compounds Analyzed by UHPLC-DAD. *protocols.io* **2024**. https://doi.org/dx.doi.org/10.17504/protocols.io.36wgqjjxyvk5/v3.

(10) Notonier, S.; Werner, A. Z.; Kuatsjah, E.; Dumalo, L.; Abraham, P. E.; Hatmaker, E. A.; Hoyt, C. B.; Amore, A.; Ramirez, K. J.; Woodworth, S. P.; Klingeman, D. M.; Giannone, R. J.; Guss, A. M.; Hettich, R. L.; Eltis, L. D.; Johnson, C. W.; Beckham, G. T. Metabolism of Syringyl Lignin-Derived Compounds in *Pseudomonas Putida* Enables Convergent Production of 2-Pyrone-4,6-Dicarboxylic Acid. *Metab. Eng.* **2021**, *65*, 111–122. https://doi.org/10.1016/j.ymben.2021.02.005.

(11) Kuatsjah, E.; Johnson, C. W.; Salvachúa, D.; Werner, A. Z.; Zahn, M.; Szostkiewicz, C. J.; Singer, C. A.; Dominick, G.; Okekeogbu, I.; Haugen, S. J.; Woodworth, S. P.; Ramirez, K. J.; Giannone, R. J.; Hettich, R. L.; McGeehan, J. E.; Beckham, G. T. Debottlenecking 4-Hydroxybenzoate Hydroxylation in *Pseudomonas Putida* KT2440 Improves Muconate Productivity from *p*-Coumarate. *Metab. Eng.* **2022**, *70*, 31–42. https://doi.org/10.1016/j.ymben.2021.12.010.
